# Pseudomonas aeruginosa Alters Peptidoglycan Composition under Nutrient Conditions Resembling Cystic Fibrosis Lung Infections

**DOI:** 10.1128/msystems.00156-22

**Published:** 2022-05-12

**Authors:** Erin M. Anderson, Neethu Shaji Saji, Alexander C. Anderson, Dyanne Brewer, Anthony J. Clarke, Cezar M. Khursigara

**Affiliations:** a Department of Molecular and Cellular Biology, University of Guelph, Guelph, Ontario, Canada; b Mass Spectrometry Facility, University of Guelph, Guelph, Ontario, Canada; c Department of Chemistry and Biology, Wilfrid Laurier University, Waterloo, Ontario, Canada; Zhejiang University School of Medicine

**Keywords:** *Pseudomonas aeruginosa*, peptidoglycan, Liverpool epidemic strain, peptidoglycomics, antibiotic resistance

## Abstract

Epidemic strains of Pseudomonas aeruginosa are highly virulent opportunistic pathogens with increased transmissibility and enhanced antimicrobial resistance. Understanding the cellular mechanisms behind this heightened virulence and resistance is critical. Peptidoglycan (PG) is an integral component of P. aeruginosa cells that is essential to its survival and a target for antimicrobials. Here, we examined the global PG composition of two P. aeruginosa epidemic strains, LESB58 and LESlike1, and compared them to the common laboratory strains PAO1 and PA14. We also examined changes in PG composition when the strains were cultured under nutrient conditions that resembled cystic fibrosis lung infections. We identified 448 unique muropeptides and provide the first evidence for stem peptides modified with O-methylation, *meso*-diaminopimelic acid (*m*DAP) deamination, and novel substitutions of *m*DAP residues within P. aeruginosa PG. Our results also present the first evidence for both d,l- and l,d-endopeptidase activity on the PG sacculus of a Gram-negative organism. The PG composition of the epidemic strains varied significantly when grown under conditions resembling cystic fibrosis (CF) lung infections, showing increases in O-methylated stem peptides and decreases in l,d-endopeptidase activity as well as an increased abundance of de-N-acetylated sugars and l,d-transpeptidase activity, which are related to bacterial virulence and antibiotic resistance, respectively. We also identified strain-specific changes where LESlike1 increased the addition of unique amino acids to the terminus of the stem peptide and LESB58 increased amidase activity. Overall, this study demonstrates that P. aeruginosa PG composition is primarily influenced by nutrient conditions that mimic the CF lung; however, inherent strain-to-strain differences also exist.

**IMPORTANCE** Using peptidoglycomics to examine the global composition of the peptidoglycan (PG) allows insights into the enzymatic activity that functions on this important biopolymer. Changes within the PG structure have implications for numerous physiological processes, including virulence and antimicrobial resistance. The identification of highly unique PG modifications illustrates the complexity of this biopolymer in Pseudomonas aeruginosa. Analyzing the PG composition of clinical P. aeruginosa epidemic strains provides insights into the increased virulence and antimicrobial resistance of these difficult-to-eradicate infections.

## INTRODUCTION

Pseudomonas aeruginosa is an opportunistic pathogen of significant concern, as it causes considerable morbidity and mortality in immunocompromised individuals. P. aeruginosa is prevalent in lung infections of people with cystic fibrosis (pwCF), with up to 70% of adult individuals infected with a strain (1). Once established, P. aeruginosa infections can be difficult to eradicate due to their ability to form recalcitrant biofilms and to develop resistance to antimicrobials (2). Of greatest concern are a group of clinical isolates called the epidemic strains (3). Isolates have been discovered in England called the Liverpool epidemic strains (LES) (3), whereas additional isolates have been found, including from Canada the LESlike isolates and the prairie epidemic strains (PES) (4, 5) as well as the Australian epidemic strains (AES) (6) and the DK2 strains from Denmark (7, 8). Unlike other clinical P. aeruginosa isolates, the epidemic strains are transmissible from patient to patient and are associated with increased virulence, higher antimicrobial resistance, and an overall worse prognosis for pwCF (4, 9, 10). Therefore, understanding the mechanisms that separate the epidemic strains from other P. aeruginosa strains is highly important.

Several recent studies have used genomic, transcriptomic, and proteomic analyses to understand global differences between the epidemic strains and other P. aeruginosa isolates (11–14). These omic techniques examine genomes, gene expression, and protein levels to predict a cellular activity that is part of key physiological processes, such as virulence and/or antimicrobial resistance. However, these techniques infer an activity *in vivo* without directly assessing the abundance of the resulting cellular components. One area where this gap has been evident is when studying the dynamics of complex biopolymers such as peptidoglycan (PG). PG is an integral structural component of bacterial cell walls and has been shown to be important for many physiological processes, including cell division, antimicrobial resistance, and virulence. The basic subunit of the PG, called a muropeptide, is a disaccharide of *N-*acetylglucosamine (NAG) and *N*-acetylmuramic acid (NAM) with a stem peptide initially consisting of 5 amino acids attached to the NAM moiety. In Gram negative bacteria, the stem peptide is typically composed of an l-alanine (position 1), d-*iso*-glutamate (position 2), *meso*-diaminopimelic acid (*m*DAP) (position 3), and two terminating d-alanine residues (positions 4 and 5), whereas in Gram positives the stem peptide composition is highly diversified, and it is the basis of a taxonomic nomenclature system (15). During synthesis, PG muropeptides are produced in the cytosol and are exported to the periplasm in Gram negatives or external to the cell in Gram positives (16, 17). Once exported, a collection of enzymes known as the penicillin-binding proteins incorporate muropeptides into the existing PG structure using transglycosylase and transpeptidase activities (18, 19). Transglycosylases produce the β-1,4-glycosidic bond between NAG-NAM repeats, creating the polysaccharide backbone of the PG, while transpeptidases generate peptide cross-linkages between the stem peptides of adjacent subunits, which stabilize and produce the mesh-like PG structure (sacculus) that surrounds the entire cell.

Once produced, PG can be modified or degraded in response to physiological pressures, such as cell division, environmental stresses, or cell signaling. For example, various lytic enzymes degrade PG during maturation and turnover (20). These enzymes include carboxypeptidases, endopeptidases, and amidases that degrade the stem peptides as well as lytic transglycosylases that lyse the polysaccharide backbone. In addition, modifications can be made to the NAG or NAM sugars, including O-acetylation, de-N-acetylation, or N-glycolylation (21, 22). Likewise, the stem peptide can be modified by, e.g., amidation or covalent attachment of proteins, and it can have variations in peptide cross-links (22). Many of these modifications to PG have been associated with important cellular processes. For example, the addition of either a d-serine or d-lactate to the terminus of the stem peptide is associated with resistance to some antimicrobials in Gram positive bacteria (23, 24).

Peptidoglycomics is a robust semiquantitative bioinformatic technique to directly identify and assess the abundance of PG components and thereby infer the enzymatic activity involved in the production and/or modification of the PG (25, 26). We recently developed a peptidoglycomic workflow that assesses the composition of the PG with a level of fine detail not typical of other methodologies (25). Here, we used our methodology to compare the PG composition of two epidemic strains, LESB58 and LESlike1, and two typical laboratory strains, PAO1 and PA14, that were clinically isolated prior to the emergence of the epidemic strains (27, 28). In addition, we examined the compositional changes that can occur during infection by growing in a medium that reflects the nutrient conditions in CF lung infections. We identified an unprecedented 448 unique muropeptides, which was significantly more than identified previously (25, 29). Differential analysis was used to assess and compare muropeptide abundance differences pairwise between each strain (strain-to-strain) and between the two growth mediums (strain-to-media). We found that all strains underwent significant remodeling of the PG when grown under conditions that mimicked the CF lung environment. In addition, we identified specific PG modifications that varied in abundance uniquely to each strain. These identified changes to the PG composition indicate functional variations between strains or growth conditions that influence virulence and antimicrobial resistance.

## RESULTS

### Production and isolation of peptidoglycan sacculi.

To assess the influence of nutrients on muropeptide composition, we cultured four strains of P. aeruginosa in either a rich nutrient medium (tryptic soy broth; TSB) or a medium that reflects the nutrient composition of CF lung infections (synthetic CF sputum medium; SCFM) (30). A comparison of growth rates showed that LESB58 and LESlike1 had longer doubling times than PAO1 and PA14 in both TSB and SCFM (see Fig. S1 in the supplemental material). Optical density values at 600 nm (OD_600_) of 0.5 represented mid-log phase for all the strains (Fig. S1) and produced consistent numbers of CFU/mL (Table S1). Only LESB58 cultured in TSB showed a significant increase in number of CFU/mL (Table S1). The quantity of peptidoglycan sacculi isolated from each strain-medium combination was variable (0.27 to 7.73 mg sacculi/g of wet cell pellet) but was not significantly different across the sampling conditions tested (Table S1).

10.1128/msystems.00156-22.1 FIG S1 Comparison of the growth of the P. aeruginosa strains over time. Each medium was inoculated from overnight-grown stationary-phase cultures. Cultures were incubated at 37°C, and the growth was monitored using the optical density at 600 nm over 24 h. (A and B) To closely mimic the growth conditions used during peptidoglycan production, 100 mL of culture was grown with shaking at 200 rpm. One milliliter of culture was taken at each time point and the OD_600_ measured. (C and D) To closely examine the differences in the growth of the four strains, growth was monitored in a 96-well spectrophotometer with 150 μL of culture layered with 50 μL mineral oil with orbital shaking. Measurements were taken every half hour. (A and C) Cells grown in TSB. (B and D) Cells grown in SCFM. Download FIG S1, TIF file, 0.8 MB. Copyright © 2022 Anderson et al. 2022 Anderson et al. https://creativecommons.org/licenses/by/4.0/ This content is distributed under the terms of the Creative Commons Attribution 4.0 International license.

10.1128/msystems.00156-22.5 TABLE S1 Culture growth and production of PG sacculi. Asterisk represents a Benjamini-Hochberg significant *P* value of <0.05. Download Table S1, XLSX file, 0.01 MB. Copyright © 2022 Anderson et al. 2022 Anderson et al. https://creativecommons.org/licenses/by/4.0/ This content is distributed under the terms of the Creative Commons Attribution 4.0 International license.

### High-resolution muropeptide mass spectrometry analysis.

**FIG 1:**
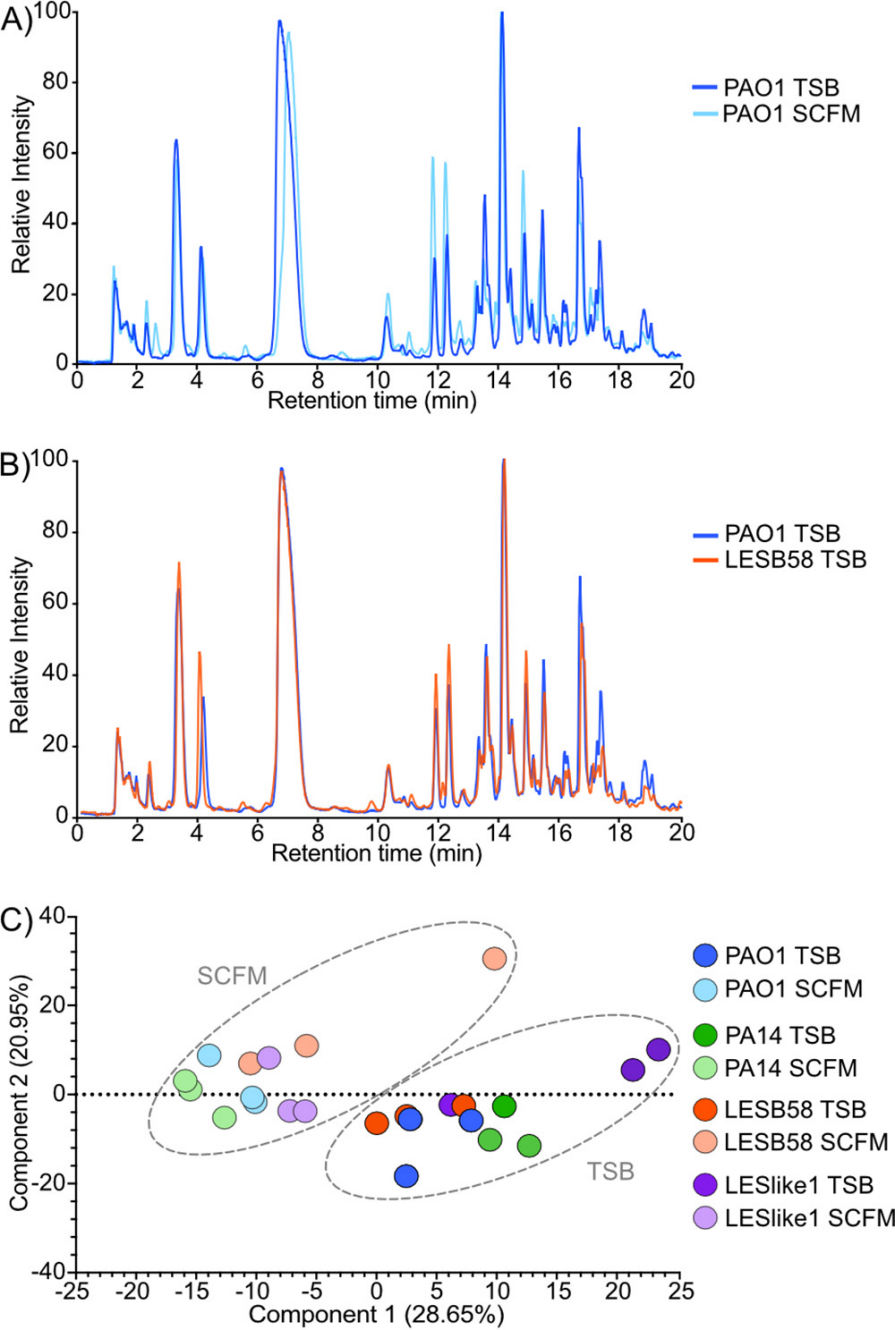
Gross peptidoglycan variations between samples. (A) The total ion chromatograph (TIC) from Q-TOF mass spectrometry of one technical replicate of PAO1 grown in TSB (dark blue) overlaid with the TIC of one technical replicate of PAO1 grown in SCFM (light blue). (B) The same TIC of PAO1 grown in TSB (dark blue) in panel A overlaid with one technical replicate of LESB58 grown in TSB (dark orange). (C) The principal-component analysis comparing all samples. Individual strains are designated by a color: PAO1 (blue), PA14 (green), LESB58 (orange), and LESlike1 (purple). The two growth media are designated by the intensity of color: TSB (dark) and SCFM (light). The gray dashed circles highlight the principal components that cluster together based on growth media.

Total ion chromatographs (TIC) of mutanolysin-digested sacculi isolated from each strain grown in either TSB or SCFM demonstrated distinct differences in muropeptide profiles (e.g., Fig. 1A). This indicates that culture conditions impact PG composition, which can be discerned by this analytical approach. When comparing strains cultured under the same conditions, such as PAO1 and LESB58 cultured in TSB, the TIC peak intensity differences were less pronounced (Fig. 1B). To determine if the observed differences represented true variations in the PG composition, a principal-component analysis (PCA) of the data set was conducted. The PCA plot indicated that the largest distinction within the data was the growth medium (component 1 at 28.65%) (Fig. 1C). There was a smaller distinction (component 2 at 20.95%) that represented strains. Overall, the PCA showed that the nutrient composition of the growth medium has a pronounced influence on the PG composition in P. aeruginosa.

To examine the apparent changes to the PG composition, we applied a liquid chromatography-tandem mass spectrometry (LC-MS/MS)-based bioinformatic methodology we developed previously (25). First, individual muropeptides were identified by comparison of the neutral *m/z* to an in-house prepared library of >170,000 muropeptide structures (Table S2). In total, we identified 448 putative muropeptides (Table S3). Of these, however, 157 did not match the *m/z* of any database entries, and therefore we removed them from the analysis. An additional 68 muropeptides were assigned a structure based on database matches but, due to very low abundance or minimal abundance variations, were not manually confirmed and hence were not included in further analysis. Overall, 223 muropeptides were annotated and manually confirmed using MS/MS (Table S3, italics and boldface), which represented 86 to 89% of the overall PG composition in each sample.

10.1128/msystems.00156-22.6 TABLE S2 Muropeptide library used for annotation of MS peaks. Download Table S2, XLSX file, 4.7 MB. Copyright © 2022 Anderson et al. 2022 Anderson et al. https://creativecommons.org/licenses/by/4.0/ This content is distributed under the terms of the Creative Commons Attribution 4.0 International license.

10.1128/msystems.00156-22.7TABLE S3List of all muropeptides identified in this study. FDR represents significance testing using Benjamini-Hochberg false discovery rate. FC represents the fold change between the indicated samples. 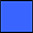 *N*-acetyl glucosamine (NAG), 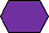 *N*-acetyl muramic acid (NAM), 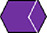 1,6 anhydro *N*-acetyl muramic acid (anh), 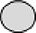 alanine (A), 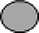 *iso*-glutamate (E), 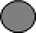 *meso*-diaminopimelic acid (*m*DAP), 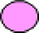 *iso*-glutamine (Q), 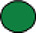 glycine (G), 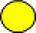 lysine (K), 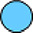 serine (S), 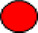 arginine (R), 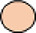 histidine (H), 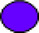 glutamate (E), 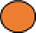 asparagine (N), 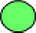 valine (V), 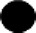 aspartate (D), 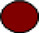 methionine (M), 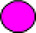 phenylalanine (F), 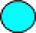 cysteine (C), 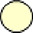 tyrosine (Y), 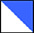 glucosamine, 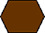 muramic acid, 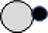 methylated alanine, 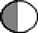 deaminated *m*DAP. Download Table S3, XLSX file, 0.8 MB.Copyright © 2022 Anderson et al.2022Anderson et al.https://creativecommons.org/licenses/by/4.0/This content is distributed under the terms of the Creative Commons Attribution 4.0 International license.

**FIG 2:**
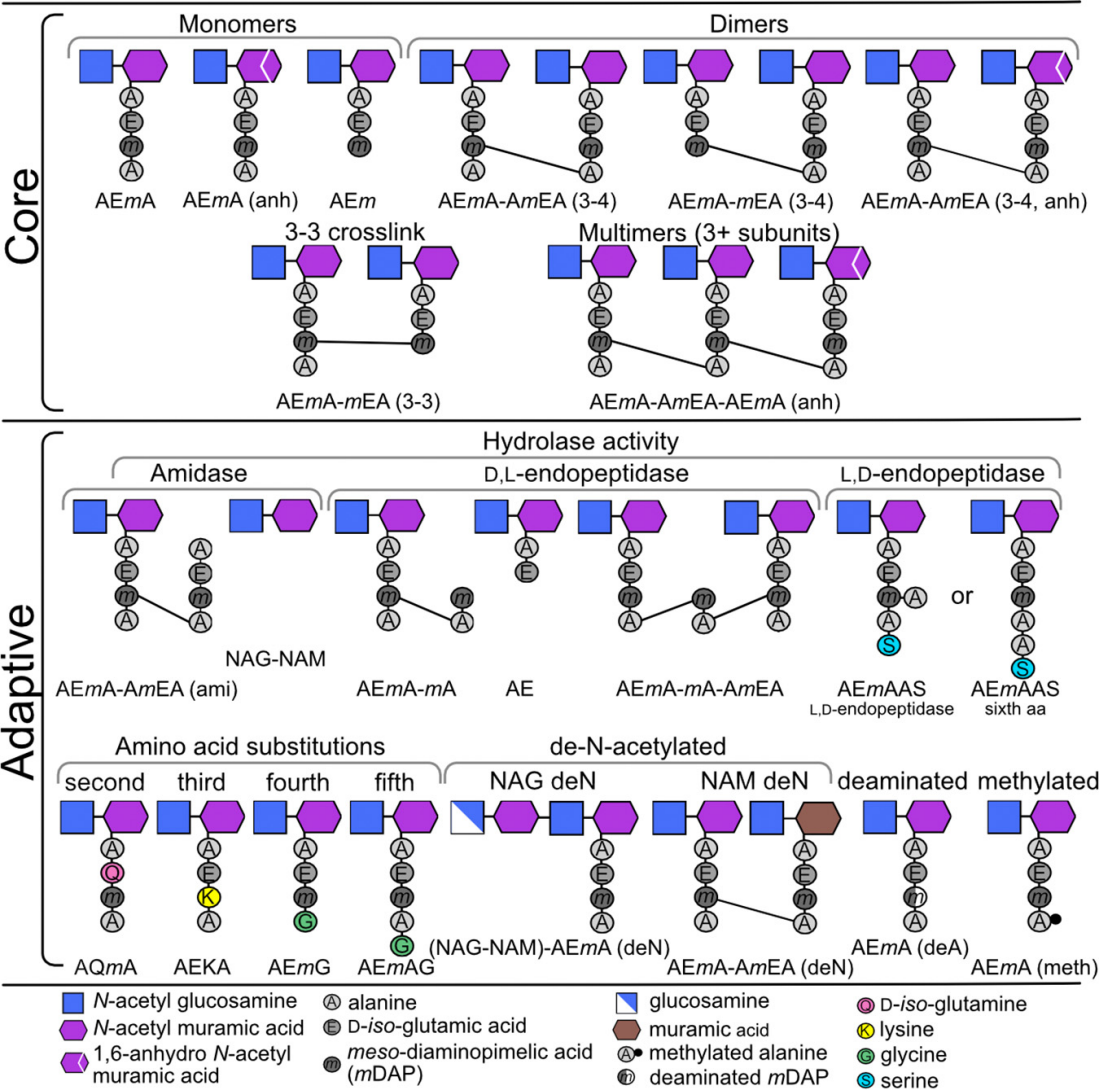
Graphical representation of the muropeptides identified in the peptidoglycan (PG) of P. aeruginosa in this study. Muropeptides were grouped as part of the core or adaptive PG. The six muropeptides at the top of the table represent the core of the PG on which modifications occurred. Modifications considered still part of the core were the change to a 3–3 cross-link and production of multimers. Adaptative PG were modifications to the core that occurred either singly or in combination.

**FIG 3:**
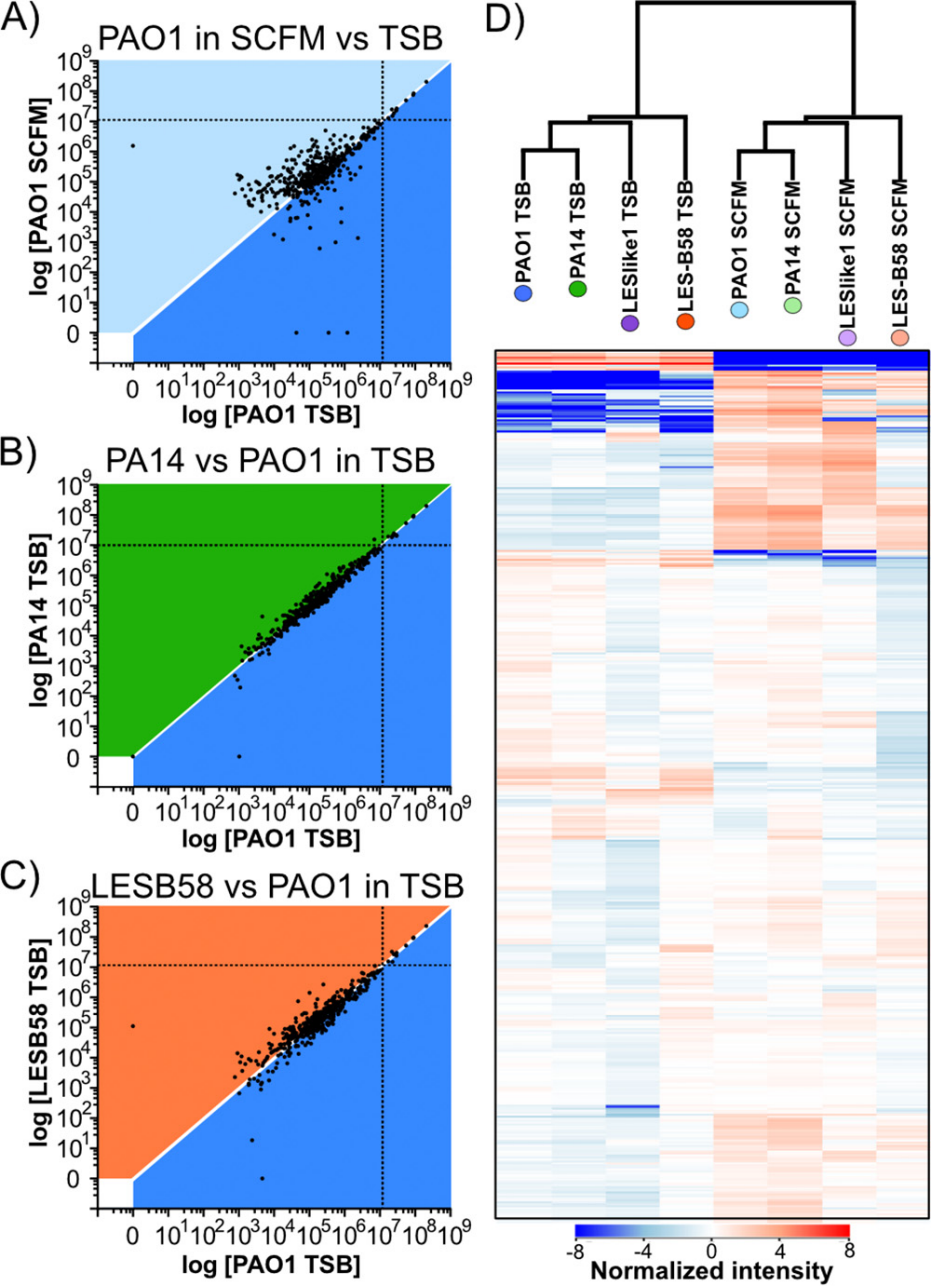
Abundance variations of muropeptides across samples. (A to C) Representative scatterplots of the log raw intensity values between samples. Each dot represents the raw intensity value of a single putative muropeptide measured within each sample plotted on the *x* and *y* axes. The white line where *x* = *y* represents intensity values that were similar in both samples. Each section is colored to represent where intensity values would represent an increase in abundance in one sample, with PAO1 grown in TSB (dark blue) compared to PAO1 grown in SCFM (light blue) (A), PA14 grown in TSB (dark green) (B), or LESB58 grown in TSB (dark orange) (C). The black vertical and horizontal dotted lines are the raw intensity value, which represents 1% of the total PG in the sample designated on the *x* and *y* axes, respectively. (D) Hierarchical clustering analysis on intensity values that were normalized to the median intensity across all samples. Red represents an increase in intensity and blue represents a decrease in intensity from the median. Each column represents a sample, and each band represents a single putative muropeptide within the sample. Muropeptides were clustered vertically in the column based on similarities in the intensity variations. Samples were then clustered based on overall muropeptide abundance variations. Additional scatterplots can be found in Fig. S2.

The four most abundant muropeptides in all samples were AE*m*A, AE*m*, AE*m*A-A*m*EA (3–4), and AE*m*A-A*m*EA (3–4, anh) (Fig. 2), which together represented 45 to 50% of the overall PG in each sample (Table S4). The monomers AE*m*A and AE*m* correspond to removal of the 5th and 4th alanine residues, respectively, from the stem peptide (Fig. 2). The dimer AE*m*A-A*m*EA (3–4) is the cross-linkage of the 4th alanine of the stem peptide to *m*DAP on a second monomer (Fig. 2), while AE*m*A-A*m*EA (3–4, anh) is the AE*m*A-A*m*EA (3–4) dimer with 1,6-anhydroNAM (anh) in place of NAM (Fig. 2). The next most abundant muropeptides each represented from 1 to 4.5% of the overall PG composition (Table S3). Within this group of ~8 to 10 highly abundant muropeptides, several modifications to the PG are represented. These include amidase activity (digesting the NAM-alanine peptide bond of the stem peptide), the addition of an amino acid other than alanine at the 5th position of the stem peptide, and the cross-linkage of three AE*m*A stems producing a trimer (Fig. 2). The remaining ~430 muropeptides are present in much lower abundance, each consisting of <1% of the overall PG (Fig. 3A to C and Fig. S3).

10.1128/msystems.00156-22.3 FIG S3 Volcano plots comparing abundance variations pairwise between samples. Each dot represents a putative muropeptide with the representative strain-to-medium comparisons between TSB and SCFM for PAO1 (A), PA14 (B), LESB58 (C), and LESlike1 (D). (E to H) Strain-to-strain comparisons for growth in TSB between PAO1 and PA14 (E), LESB58 (F), and LESlike1 (G). (H) Strain-to-strain comparison of LESlike1 compared to LESB58 in TSB. (I to L) Strain-to-strain comparisons for growth in SCFM between PAO1 and PA14 (I), LESB58 (J), and LESlike1 (K). (L) Strain-to-strain comparison of LESlike1 compared to LESB58 when grown in SCFM. Each quadrant is colored to represent the sample where the muropeptide abundance is increased. The dotted vertical lines represent a 2-fold change in abundance. The dotted horizontal line represents a significant adjusted *P* value (FDR) of 0.05. Download FIG S3, TIF file, 1.5 MB. Copyright © 2022 Anderson et al. 2022 Anderson et al. https://creativecommons.org/licenses/by/4.0/ This content is distributed under the terms of the Creative Commons Attribution 4.0 International license.

10.1128/msystems.00156-22.8 TABLE S4 The abundance differences and the 1D annotation for specific muropeptide characteristics. The categories labeled as combined represent all the muropeptides that had the specific structure but could also contain structures that indicate additional modification, whereas the remaining categories represent muropeptides with only structures for a single modification on the core PG structure. FC represents the fold change between the indicated samples. Score is the 1D annotation score where closer to 1.0 indicates strong enrichment and scores closer to 0 indicate no enrichment. FDR is the Student’s *t* test that was corrected using the Benjamini-Hochberg false discovery rate on the 1D annotation category. Boldfaced green highlight represents 1D annotation scores that were significant with FDR of <0.05. Blue highlighting represents categories that contained only 1 or 2 muropeptides and significance testing could not be performed. Download Table S4, XLSX file, 0.1 MB. Copyright © 2022 Anderson et al. 2022 Anderson et al. https://creativecommons.org/licenses/by/4.0/ This content is distributed under the terms of the Creative Commons Attribution 4.0 International license.

10.1128/msystems.00156-22.2 FIG S2 Scatterplots of the MS raw intensity values. Each dot represents the raw intensity value of a putative muropeptide within each sample represented on the *x* and *y* axes. (A to D) are strain-to-medium comparisons with growth in TSB on the *x* axis and SCFM on the *y* axis for PAO1 (A), PA14 (B), LESB58 (C), and LESlike1 (D). (E to H) Strain-to-strain comparisons for growth in TSB between PAO1 and PA14 (E), LESB58 (F), and LESlike1 (G). (H) Strain-to-strain comparison of LESlike1 and LESB58 in TSB. (I to L) Strain-to-strain comparisons for growth in SCFM between PAO1 and PA14 (I), LESB58 (J), and LESlike1 (K). (L) Strain-to-strain comparison of LESlike1 compared to LESB58 when grown in SCFM. The dotted lines are the raw intensity value that represents 1% of the PG in the sample. Areas are colored to illustrate the sample where a muropeptide would have an increased abundance. Download FIG S2, TIF file, 1.7 MB. Copyright © 2022 Anderson et al. 2022 Anderson et al. https://creativecommons.org/licenses/by/4.0/ This content is distributed under the terms of the Creative Commons Attribution 4.0 International license.

Next, differential analysis was used to assess any variations in abundance of the muropeptides across the samples tested. To reduce the influence of the highly abundant muropeptides, the intensity of each muropeptide was normalized to the median intensity across all samples. Hierarchical clustering analysis was used to group the data and determine gross similarities or differences. Within each sample, hierarchical clustering of muropeptides indicated the presence of distinct sets that varied substantially from the normalized median intensity (Fig. 3D). This indicated that groups of muropeptides did vary considerably and distinctly within each sample. When hierarchical clustering was compared between the samples, the samples clustered most distantly based on the growth medium (Fig. 3D). However, within each growth medium PAO1 clustered the closest with PA14 and LESB58 had the most distant clustering of the four strains (Fig. 3D). Overall, this indicated that PG composition was the most similar between PAO1 and PA14 and the most distinct between the growth media.

**FIG 4:**
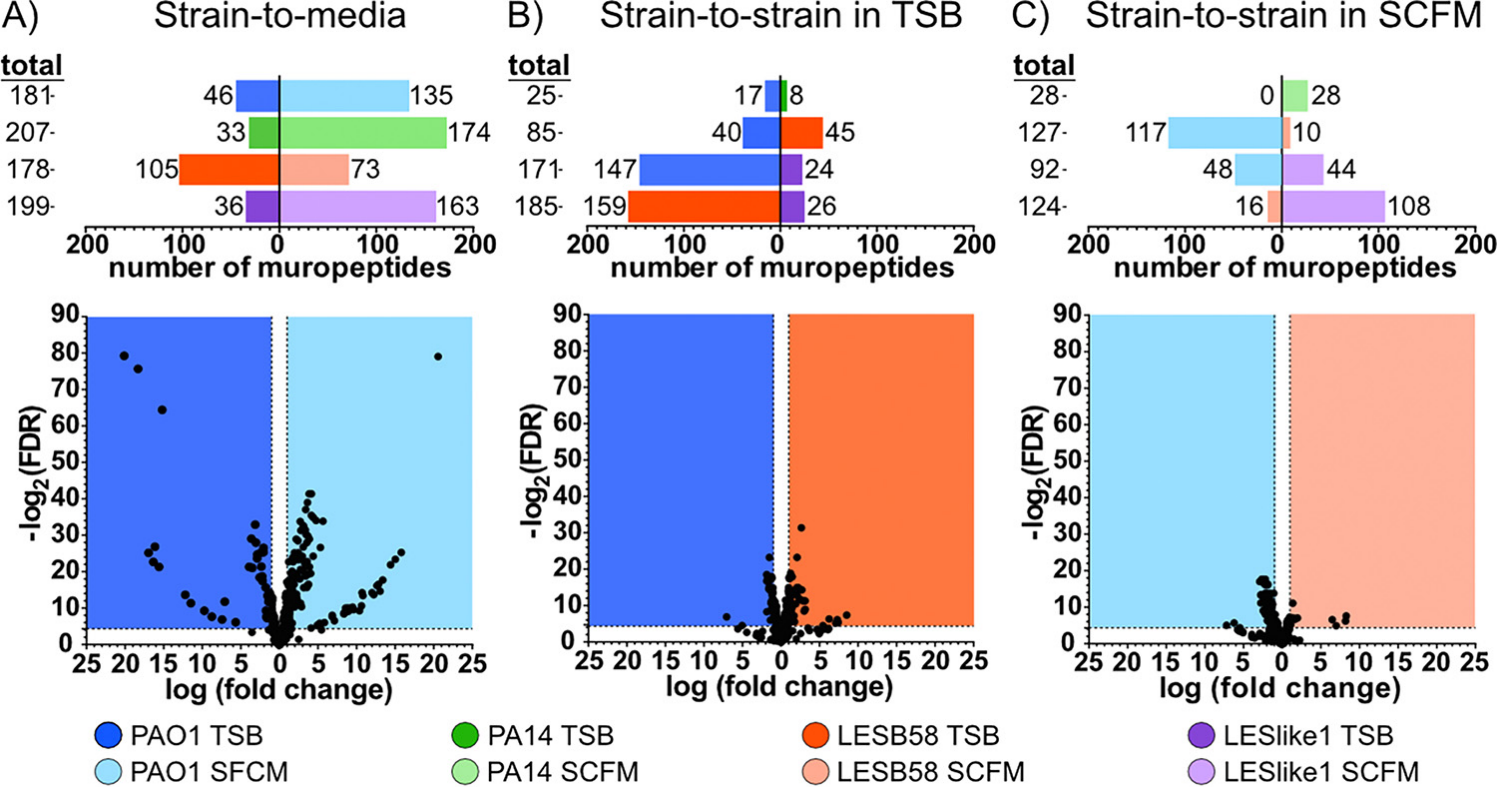
Comparison of muropeptide variation across samples. Volcano plot comparisons were conducted pairwise between samples to illustrate peptidoglycan variations that occurred due to growth media (A) or occurred between strains when grown in TSB (B) or SCFM (C). (Top) Number of muropeptides that had a significantly high fold change (FDR < 0.05; FC > 2) between two samples. The total numbers of muropeptides are listed on the left side of the panel, whereas the bars indicate the numbers of muropeptides that were increased in abundance in each sample. The bottom panel is a representative volcano plot used to produce the top panel, illustrating the level of FDR and FC for each putative muropeptide (dot). Additional volcano plots can be found in Fig. S3.

Each strain-to-strain and strain-to-medium combination was further evaluated for muropeptide abundance variations, and the significance was determined using a Student's *t* test that was corrected using the Benjamini-Hochberg false discovery rate (FDR) (31). The strain-to-medium comparison between growth in TSB and SCFM produced the greatest number of muropeptides (total of between 178 and 207) that varied with a significantly high (>2) fold change (FC) (Fig. 4A). Volcano plots of these strain-to-medium combinations demonstrated a dispersed scatter pattern with up to 15- to 20-fold changes in abundance (Fig. 4A and Fig. S3). This supports the PCA, indicating the growth medium has a profound effect on PG composition.

In strain-to-strain comparisons in either TSB (Fig. 4B) or SCFM (Fig. 4C), fewer muropeptides surpassed the significant >2-fold change in abundance. In addition, the volcano plots of the strain-to-strain comparisons showed these muropeptides had <10-fold changes in abundance (Fig. 4B and C and Fig. S3). This again indicated that although the growth medium had the greatest influence on PG composition, strain-specific variations were still observed, albeit with fewer muropeptides and with lower fold changes. In addition to this, in either TSB or SCFM, PAO1 and PA14 contained the most similar PG composition, with only 25 (Fig. 4B) or 28 (Fig. 4C) total muropeptides that differed with a significant fold change of  ≥2 between the two strains. The LES strains had larger numbers of muropeptides with a significant ≥2-fold change, but they differed depending on the growth medium. For example, LESB58 had the most muropeptides (127) that significantly varied in abundance compared to PAO1 when grown in SCFM (Fig. 4C), whereas LESlike1 had the most muropeptides (171) that were significantly varied compared to PAO1 when grown in TSB (Fig. 4B). Together, this supports the hierarchical clustering, indicating the two laboratory strains have similar PG compositions, whereas the epidemic strains have more variation in their PG composition, and that variation is influenced by the growth medium.

**FIG 5:**
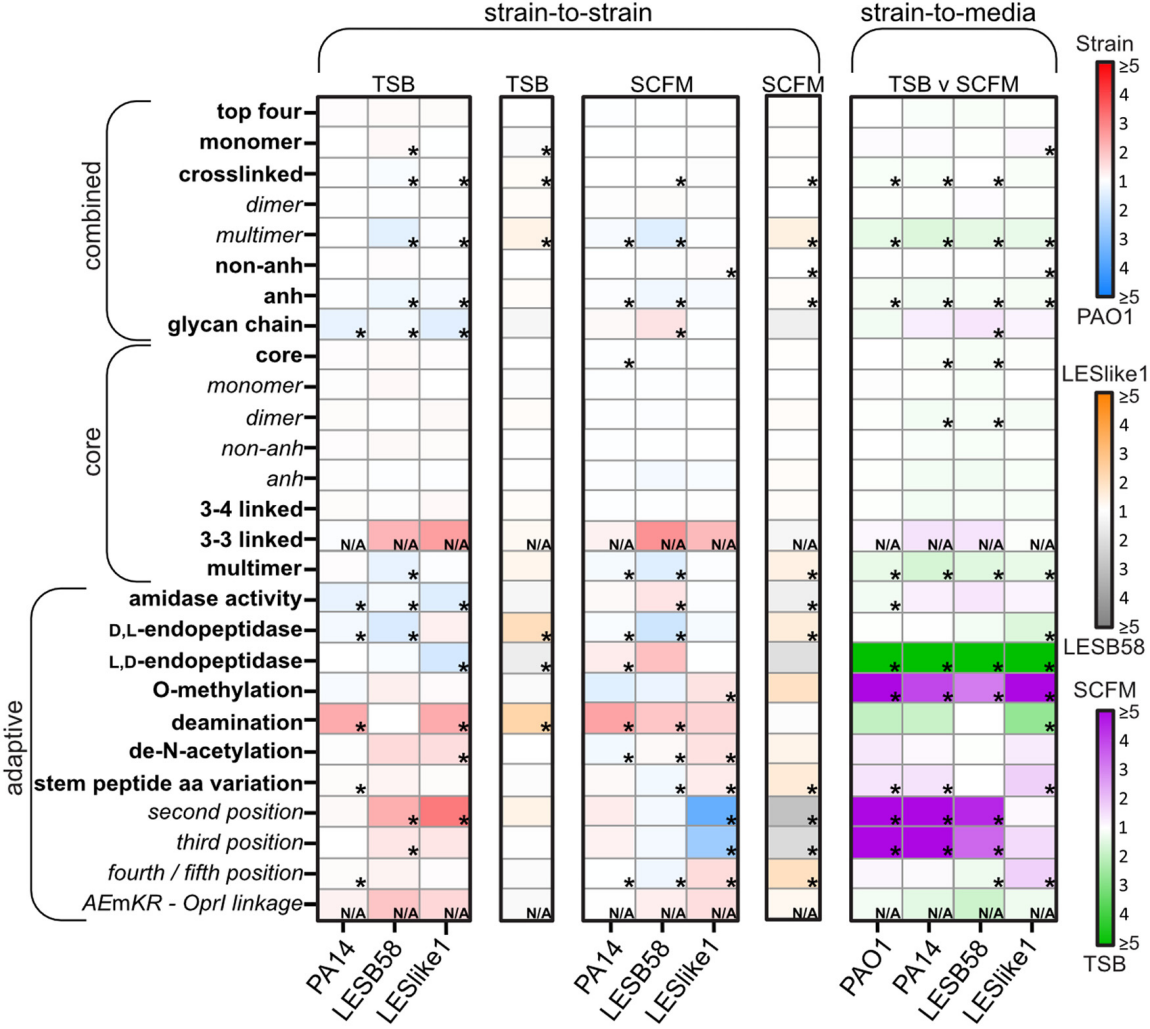
Heat map of the abundance fold changes between strain-to-strain and strain-to-medium conditions. Each box represents a group of muropeptides that contain the specified modification indicated on the left. Within the strain-to-strain comparisons, red to blue represents the comparison of each strain (red) indicated at the bottom to PAO1 (blue), whereas gold to silver represents the comparison between LESB58 (gold) and LESlike1 (silver). For the strain-to-medium comparison, green to purple represents the comparison of growth in SCFM (purple) and TSB (green) medium within each strain indicated at the bottom. The asterisk represents a significant 1D annotation for that group of muropeptides (FDR < 0.05, s0 = 1). N/A represents a group with only 1 to 2 muropeptides; therefore, significance could not be assessed with 1D annotation.

We next considered each muropeptide for the presence of structural characteristics, which indicated specific enzymatic activity on either the disaccharide or the peptide side chain. These characteristics were compiled into categories and assessed for deviation from the global distribution across all 448 muropeptides using 1D annotation and evaluated for significance using the Student's *t* test with FDR correction (31, 32). This analysis revealed enzymatic activity that differed significantly between each strain-to-strain or strain-to-medium combination (Fig. 5 and Table S4). Based on these characteristics and the identified muropeptide structures (Fig. 2), the composition of the PG can be separated into core and adaptive muropeptides (25).

### Differential analysis of the core peptidoglycan.

As noted above, four muropeptides (AE*m*A, AE*m*, AE*m*A-A*m*EA [3–4], and AE*m*A-A*m*EA [3–4, anh]) (Fig. 2) represented the largest percentage of the PG composition. Based on 1D annotation, these four muropeptides did not change in abundance (FC of ~1.0 and FDR > 0.05) between any strain-to-strain or strain-to-medium combination (Fig. 5 and Table S4). Of all 223 annotated muropeptides, 18 were identified that consisted of AE*m*A and/or AE*m* as a monomer or dimer, with or without the 1,6-anhdyro modification. These 18 muropeptides also did not have any structures resulting from any additional enzymatic activity shown in Fig. 2. Therefore, these muropeptides were designated the core PG. Using 1D annotation, these core muropeptides did not surpass the FDR < 0.05 significance threshold within most of the strain-to-strain or strain-to-medium combinations (Fig. 5 and Table S4), indicating that the core PG was relatively consistent regardless of strain or nutrient conditions. The few exceptions were the strain-to-medium comparisons for PA14 and LESB58 that were enriched in core muropeptides in TSB compared to growth in SCFM (Table S4). In SCFM, PAO1 also was enriched in the core muropeptides compared strain-to-strain with PA14 (Table S4). Although these conditions resulted in significant enrichment (FDR < 0.05) using 1D annotation, the fold change between the conditions was relatively small (FC = 1.05 to 1.13). However, due to the overall high abundance of the core muropeptides, a fold change of >2 would be unlikely. Therefore, to better understand these observed differences in the core, the 18 muropeptides were further separated into additional subcategories and reassessed using 1D annotation. These results indicated that the enrichment of core muropeptides seen in LESB58 when grown in TSB was due to the increased abundance of dimer AE*m*A-*m*EA (FC = 1.82) (Table S4), whereas the dimer AE*m*A-A*m*EA was responsible for the enrichment of the core muropeptides in PA14 grown in TSB (FC = 1.18) and for PAO1 compared to PA14 in SCFM (FC = 1.09) (Table S4). Interestingly, grouping the core muropeptides to examine specifically the 3–4 cross-link indicated no significant 1D enrichment under any of the conditions (Table S4). This suggests the presence of AE*m*A or AE*m* has more influence on the enrichment of these core dimers than the 3–4 cross-link. This preference for stem peptide length may reflect the activity of a specific penicillin-binding protein and/or l,d-carboxypeptidase that trims the stem peptide.

In addition to the 3–4 linkage, which is produced via d,d-transpeptidase activity (Fig. 2), l,d-transpeptidases can produce a 3–3 linkage between the *m*DAP residues of two adjacent stem peptides (Fig. 2). Of the 223 muropeptides, only two were confirmed to have a 3–3 linkage between AE*m*A and/or AE*m* (namely, AE*m*A-*m*EA [3–3] and AE*m*-*m*EA [3–3]). Although three other muropeptides with 3–3 cross-link were found, they also contained additional PG modifications, as shown in Fig. 2. The enzymatic activity to produce these additional modifications would influence the abundance of these muropeptides within the PG composition and, hence, could impact the differential analysis. Therefore, only muropeptides with one modification were used for 1D annotation. The abundance of the 3–3 linked muropeptides displayed a high fold change (FC > 2.0) compared to LESB58 or LESlike1 strain-to-strain with PAO1 in either TSB or SCFM (Fig. 5 and Table S4). Unfortunately, 1D annotation requires a minimum of three muropeptides to perform statistical analysis. Therefore, each muropeptide was examined individually to determine significance. Comparing LESB58 to PAO1, both AE*m*A-*m*EA (3–3) and AE*m*-*m*EA (3–3) were significantly increased >2-fold in TSB, whereas in SCFM, only AE*m*A-*m*EA (3–3) was significantly increased in abundance (Table S3). Comparing LESlike1 to PAO1 in SCFM, both muropeptides were significantly enriched, but when cultured in TSB neither muropeptide surpassed significance, with an FDR of >0.05 (Table S3). This suggests that compared to PAO1, both LESB58 and LESlike1 have increased abundance of the 3–3 cross-link when grown in SCFM, but only LESB58 significantly increased the 3–3 cross-linked muropeptides when grown in TSB.

In addition to dimers, the monomers AE*m*A and AE*m* can also be linked into larger complexes (Fig. 2). In this study, we identified multimers, including trimer (3), tetramer (4), and hexamer (6) subunits (Table S3). In total, 37 muropeptides were identified as containing a multimeric (3+) assemblage, of which 22 contained multiple modifications, as described for Fig. 2. Of the remaining 15 multimeric muropeptides, all four strains had a significant strain-to-medium increased enrichment in TSB with a fold change of 1.35 to 1.67 (Fig. 5 and Table S4). In the TSB strain-to-strain comparisons, only PAO1 was significantly enriched in multimeric muropeptides compared to LESB58 (FC = 1.38). With comparison of strains from SCFM cultures, PAO1 was enriched in multimeric muropeptides compared to both LESB58 (FC = 1.52) and PA14 (FC = 1.18) (Fig. 5 and Table S4). In addition, LESlike1 was significantly enriched in multimers compared to LESB58 (FC = 1.42) (Fig. 5 and Table S4). In summary, while significant strain-specific enrichments were evident, all the strains produced PG with increased abundance of multimeric muropeptides when cultured in TSB. Examining the characteristics of these multimers, those composed of all AE*m*A subunits and the anh modification were responsible for the observed multimer enrichment described above (Table S4). The increased abundance of the anh modification on multimeric muropeptides implies that these large, cross-linked complexes occur often at polysaccharide chain terminations.

### Differential analysis of the adaptive peptidoglycan.

We recently proposed that adaptive PG is composed of muropeptides that are present in lower quantities and may play a physiological role other than for maintenance of structural support (25). Here, we identified muropeptides indicative of amidase and endopeptidase activity. We also identified muropeptides that were deacetylated, methylated, and deaminated and muropeptides that contained amino acid variations to the typical sequence of the stem pentapeptide. Additionally, we identified muropeptides that contained structures that indicated combinations of multiple enzymes that modified a single muropeptide (Table S3).

### Hydrolase activity on mature peptidoglycan.

Muropeptides containing structures that are indicative of amidase activity that hydrolyzes the NAM-alanine linkage (Fig. 2 and Fig. S4) were the most common found within the adaptive PG. Overall, 62 muropeptides would have resulted from amidase digestion, and these muropeptides made up approximately 14% of the total PG composition (Table S4). Amidase activity was reflected in monomers, dimers, and multimers as well as in combination with all other PG modifications except methylation. Nineteen of these muropeptides would have been generated exclusively by amidase activity (Table S4), and these were used for 1D analyses. The strain-to-medium comparisons reflected an increased amidase activity in PAO1 when grown in TSB (FC = 1.21), whereas the other strains did not significantly change their amidase activity when cultured in TSB or SCFM. Comparing strain-to-strain in TSB, PAO1 showed evidence of significant enrichment of amidase activity compared to PA14, LESB58, and LESlike1 (FC = 1.39, 1.18, 1.54, respectively) (Fig. 5 and Table S4). However, this trend differed when comparing the PG from SCFM cultures, where amidase evidence was significantly enriched in LESB58 compared to PAO1, PA14, and LESlike1 (FC = 1.42, 1.31 [data not shown], and 1.52, respectively) (Fig. 5 and Table S4). This indicated that growth in TSB resulted in a significant increase in amidase activity in PAO1, whereas growth in SCFM resulted in increased amidase activity in LESB58 compared to the other strains. Amidase digestion produces both a NAG-NAM polysaccharide and a cross-linked stem peptide (Fig. 2). Examining the 19 muropeptides for both characteristics and reassessing using 1D annotation indicated that the enrichment in the PG isolated from LESB58 cultured in SCFM was due to non-anh-containing polysaccharides (FC = 1.45 to 1.57) (Table S4). With PAO1 grown in TSB we found an increased abundance of the polysaccharide, the cross-linked dimer, and both anh and non-anh muropeptides (FC = 1.15 to 1.65) (Table S4). This suggests the increased abundance within LESB58 in SCFM is the result of the increased activity of a specific amidase, whereas the increased abundance within PAO1 in TSB is the result of the increased activity of multiple amidases.

10.1128/msystems.00156-22.4 FIG S4 Example MS/MS annotation of selected adaptive PG muropeptides. Each page represents one muropeptide, indicated in the top left. At the top is the ChemDraw PG structure where the red dashed lines represent predicted fracture sites with the resulting *m/z* of the fragments indicated. At the bottom is an example MS/MS chromatogram with the associated peaks highlighted with the blue arrows and the observed *m/z* indicated. Download FIG S4, PDF file, 0.9 MB. Copyright © 2022 Anderson et al. 2022 Anderson et al. https://creativecommons.org/licenses/by/4.0/ This content is distributed under the terms of the Creative Commons Attribution 4.0 International license.

In addition to amidase activity, we also identified two additional hydrolases. The first was a d,l-endopeptidase that hydrolyzes the glutamate-*m*DAP peptide bond (Fig. 2 and Fig. S4). In total, 18 muropeptides contained structures indicative of d,l-endopeptidase activity on dimers, trimers, or tetramers, of which 9 muropeptides contained a single PG modification (Table S4). Unlike the other strains, LESlike1 showed a significant enhancement of d,l-endopeptidase activity when grown in TSB (FC = 1.54) (Fig. 5 and Table S4). Strain-to-strain comparisons indicated the d,l-endopeptidase activity was significantly increased in PAO1 and LESlike1 cultured in either TSB or SCFM compared to PA14 and LESB58, with a fold change of 1.11 to 2.02 (Fig. 5 and Table S4). Overall, this indicated that each strain produced a strain-specific level of d,l-endopeptidase activity that was only minimally affected by the nutrient composition of the growth media, except for LESlike1, which significantly increased the d,l-endopeptidase activity in TSB.

We also identified muropeptides with the peptide side chain sequence AE*m*AAx (Fig. 2 and Fig. S4). The terminal amino acid (x) was either asparagine, glycine, serine, or valine. These AE*m*AAx peptides were likely the result of l,d-endopeptidase activity (Fig. 2) that hydrolyzes the *m*DAP-alanine bond on 3–4 cross-linked dimers. These AE*m*AAx stem peptides were either not found or only very minimally present in the PG of cells when grown in SCFM. In contrast, their presence was significantly increased up to 0.22% (FC = 18 to 40) of the PG composition when cells were grown in TSB (Fig. 5 and Table S4). In addition, the TSB strain-to-strain comparison of PAO1 or LESB58 showed a significant enrichment of AE*m*AAx muropeptides compared to LESlike1 (FC = 1.73 and 1.58, respectively) (Fig. 5 and Table S4).

### Amino acid substitutions/modifications on the stem peptide.

The most diverse set of muropeptide modifications/substitutions to the stem peptide occurred at positions 2, 3, 4, and 5. In position 2, amidation of the glutamate producing a glutamine (Fig. 2 and Fig. S4) was identified in a total of 6 muropeptides. However, three of these were products of additional hydrolase activity and were removed from subsequent 1D analysis. The strain-to-medium comparison showed the PG of PAO1, PA14, and LESB58 displayed an enrichment of the glutamate amidation when cells were grown in SCFM (FC = 12.21, 14.49, and 4.49, respectively) (Fig. 5 and Table S4). Compared within SCFM, PAO1, PA14, and LESB58 were all enriched in glutamine compared to LESlike1 (FC = 3.55, 1.55 [data not shown], and 2.95, respectively) (Fig. 5 and Table S4). In contrast, for comparisons of strain-to-strain in TSB, LESB58 and LESlike1 showed an increased abundance of glutamine compared to PAO1 (FC = 2.26 and 3.11) (Fig. 5 and Table S4). Overall, these data indicated that the two laboratory strains drastically changed the glutamate amidation activity depending on growth condition, whereas LESB58 varied the activity to a lesser degree. In contrast, LESlike1 did not vary the abundance of glutamate amidation under the conditions tested.

The addition of a unique amino acid (other than alanine) at the terminus of the stem peptide (those at position 4 or 5; Fig. 2 and Fig. S4) represented the second most abundant modification within the adaptive PG (behind amidase activity), constituting 7 to 13% of the overall PG composition (Table S4). In total, 91 muropeptides were found with either arginine, asparagine, aspartate, cysteine, glycine, histidine, leucine, lysine, methionine, phenylalanine, serine, tyrosine, or valine incorporated at either the 4th (AE*m*x) or 5th (AE*m*Ax) position of the stem peptide sequence (Table S4). Muropeptides with AE*m*x or AE*m*Ax variations were found as monomers, dimers, and trimers and in conjunction with amidase and d,l-endopeptidase activity. In total, 71 muropeptides were found without any additional multimeric or hydrolase activity. With strain-to-medium comparisons, LESlike1 showed increased abundance when cultured in SCFM (FC = 1.73), whereas LESB58 showed enrichment when grown in TSB (FC = 1.27) (Fig. 5 and Table S4). With the strain-to-strain comparison of cells grown in SCFM, LESlike1 was enriched compared to the other strains (FC = 1.55 to 1.96) (Table S4). Similarly, in SCFM PAO1 was significantly enriched compared to both PA14 and LESB58 (FC = 1.03 and 1.26) (Table S4). In contrast, with TSB strain-to-strain comparisons, PA14 was significantly enriched compared to PAO1 and LESlike1 (FC = 1.06 and 1.02 [data not shown]); however, the fold change was minimal. Altogether these data indicated the medium composition influenced the addition of unique amino acids at the terminus of the stem peptide differently in the four strains. Cultured in SCFM, LESlike1 produced the highest abundance of the terminal amino acid variations and LESB58 contained the lowest abundance. AE*m*Ax was preferred over AE*m*x, which constituted ~6% versus ~2% of overall PG composition, respectively (Table S4). In strain-to-medium comparisons, LESB58 preferentially increased AE*m*Ax when grown in TSB (FC = 1.37), while in SCFM, PAO1 preferentially increased AE*m*x (FC = 1.67) and LESlike1 enriched both AE*m*x (FC = 1.64) and AE*m*Ax (FC = 1.77) (Table S4). This indicates that each strain varied the addition of the terminal amino acids in either the 4th or 5th position differently in each growth medium.

### Identification of unique muropeptides.

**FIG 6:**
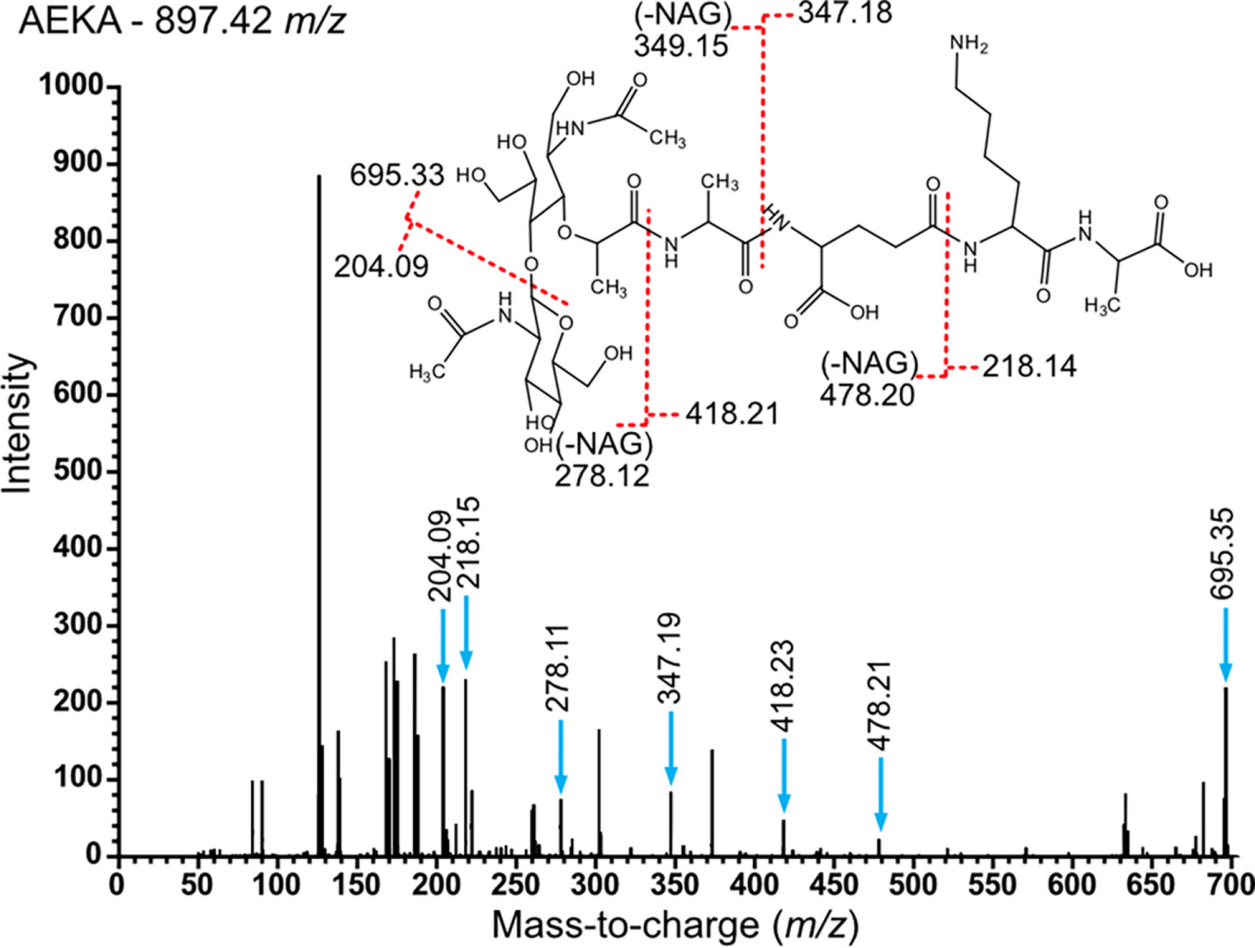
Example MS/MS spectrum for the annotation of the putative muropeptide AEKA. (Inset) The MS/MS spectra were compared to the predicted fragmentation of the AEKA structure. The predicted *m/z* fragments resulting from breakage occurring at the red dotted lines on the inset were compared to the peaks found in the MS/MS spectra (blue arrows). An additional spectrum is in Fig. S4.

One of the defining features of Gram negative PG is the presence of an *m*DAP residue at position 3 of the stem peptide (Fig. 2). However, in this study we identified amino acid substitutions of the 3rd residue consisting of alanine, aspartate, glutamate, glycine, lysine, serine, or valine (Table S3). This variation of the 3rd amino acid was most often appended with a terminal alanine residue (AExA) (Fig. 2 and 6 and Fig. S4). Muropeptides with a 3rd position substitution were found cross-linked in a dimer or trimer (Table S3). Overall, 23 muropeptides contained an alternate 3rd amino acid, of which 8 also contained structures representing additional enzymatic activity. The remaining 15 muropeptides with a 3rd position substitution demonstrated a significantly high fold enrichment when grown in SCFM for PAO1, PA14, and LESB58 (FC = 3.43 to 6.86) (Fig. 5 and Table S4). Comparing strain-to-strain in SCFM, PAO1, PA14, and LESB58 showed a significant increase in the 3rd position substitution compared to LESlike1 (FC = 2.61, 3.11 [data not shown], and 2.18, respectively) (Fig. 5 and Table S4). Together these data indicated that LESlike1 did not demonstrate a medium-derived change in abundance of the 3rd position substitutions like the other strains.

### Additional modifications of the muropeptide.

Three additional modifications were identified that included de-N-acetylation (deN) of the polysaccharide as well as methylation and deamination of the stem peptide (Fig. 2 and Fig. S4). There were only 5 muropeptides annotated that contained a deN sugar moiety, 1 muropeptide with a deN on the NAM and 4 muropeptides with deN modification of the NAG, which were also always in conjunction with amidase activity (Fig. 2 and Table S3). We did not identify any strain-to-medium differences; however, several strain-to-strain differences were noted. LESlike1 demonstrated an enrichment of the NAG deN modification in either medium compared to PAO1 (FC = 2.27 and 2.06) and PA14 (FC = 2.98 and 2.71, data not shown) (Fig. 5 and Table S4). In the strain-to-strain comparison in SCFM, LESB58 was enriched in the deN muropeptides compared to PAO1 (FC = 1.54) and PA14 (FC = 2.03, data not shown). In addition, PAO1 was significantly more abundant in deN modifications than PA14 (FC = 1.32) (Fig. 5 and Table S4). Therefore, the NAG deN modification demonstrated a strain-specific enrichment where the epidemic strains had the highest abundance and PAO1 was slightly higher in abundance than PA14. Interestingly, this strain-specific deN enrichment does not mirror the strain-to-strain variation of amidase activity. This indicates that deN enzymatic activity on the NAG occurred after amidase digestion.

A structure indicating putative O-methylation was found on 3 muropeptides and occurred on the terminal alanine of the stem peptide, as determined using the MS/MS fragmentation profile (Table S3 and Fig. S4). These methylated muropeptides were significantly enriched in SCFM in strain-to-medium comparisons in all strains (FC = 3.11 to 6.86) (Fig. 5 and Table S4). Minimal strain-to-strain variation was seen with only LESlike1 in SCFM enriched in methylated muropeptides compared to PAO1 (FC = 1.44) (Fig. 5 and Table S4).

Finally, a putative deamination (deA) was found on 6 muropeptides (Table S3). Deamination was determined to be on the *m*DAP by MS/MS fragmentation analysis (Fig. S4). Additionally, deA was commonly found in conjunction with amidase activity (4 of the 6 muropeptides), and the dual modification represented a higher proportion of the PG composition, ~1% to 2% versus ~0.04% without amidase activity (Table S4). In the strain-to-medium comparisons, only LESlike1 significantly increased the abundance of deA muropeptides when grown in TSB (FC = 2.68) (Fig. 5 and Table S4). This resulted in LESlike1 in TSB having significantly enriched deA muropeptides compared strain-to-strain with PAO1 or LESB58 (FC = 2.32 and 2.32) (Fig. 5 and Table S4). In addition, PA14 had significantly more deA muropeptides than PAO1 in both TSB (FC = 2.32) and SCFM (FC = 2.44) (Fig. 5 and Table S4), whereas LESB58 was only enriched in deA compared to PAO1 when grown in SCFM (FC = 1.89) (Fig. 5 and Table S4). Therefore, LESlike1 was influenced the most by the medium composition to vary the abundance of the deA muropeptides, whereas PA14 had a strain-specific higher abundance of the deA muropeptides and LESB58 was not significantly influenced by the media.

## DISCUSSION

The method for purification of PG sacculi has remained relatively unchanged since the 1960s (33), while similarly the method for compositional analysis based on reverse-phase high-performance liquid chromatography (HPLC) has remained the standard protocol since it was first developed by Glauner in the 1980s (34). However, recently the application of newer technologies to enhance and streamline the analysis methodology has been proposed and advanced (25, 35–38). Our previous study demonstrated the application of MS feature extraction coupled with a bioinformatic methodology proved to be useful in examining the composition of the PG from P. aeruginosa at a level of detail not seen using other methodologies (25). Patel et al. (38) recently developed a novel piece of software, PGfinder, which is a robust, open-access platform for the automated annotation of muropeptides. The comparison of the muropeptide annotations from our previous study aligned well with the annotation performed by Patel et al. using PGfinder (38).

To extend our previous findings comparing planktonic and biofilm growth morphologies, we used our bioinformatics methodology to compare the PG from both laboratory-derived and epidemic strains of P. aeruginosa. In addition, we examined the effect of growth conditions on PG composition by comparing a rich nutrient medium and a medium that simulates the nutrient conditions found within CF lung infections. Using SCFM to replicate aspects of CF lung infections provides a good model for assessing the physiology of P. aeruginosa during infection (39, 40). Here, we identified 448 unique muropeptide structures present at the mid-exponential phase of growth. Previously when examining 96-h stationary-phase PAO1 cells, we identified 160 unique muropeptides using this same methodology (25). This difference in the total number of identified muropeptides between these two experiments indicates that PG composition in P. aeruginosa is more dynamic in growing cells and may become less varied as cultures age. It was well known that PG composition will change as bacteria enter stationary phase and age (41–43), but it was not previously noted that its composition becomes less diverse.

As in our previous study, we were able to designate a core PG composition that was highly stable between the different physiological states of either biofilm or planktonic cell growth (25). Here, we found this core PG to also be highly stable across multiple P. aeruginosa strains and when grown under the different nutrient conditions tested. This indicates that the multiple PG synthesis/degradation enzymes concurrently function to produce a relatively stable core PG composition that may serve as the structural framework of the PG. The stability of this core PG and the relatively high abundance of the core muropeptides, coupled with the use of less sensitive methodologies, may explain why the composition of Gram negative PG has often been considered to be relatively constant (15, 44). Within the core PG, we did identify minimal variation within dimers between the conditions tested, which indicates a slight change in the activity of just a few PG synthetic enzymes.

Although we did not include muropeptides consisting of multimers and 3–3 cross-links as part of the core PG throughout our bioinformatics analysis, we have still included them under the core subheading due to their participation in overall PG structure and cell wall stability. The production of the 3–3 cross-link is a well-known bypass mechanism that can compensate for the loss of the 3–4 cross-link when d,d-transpeptidase activity is blocked by β-lactam antimicrobial treatment (45, 46). The increased abundance of the 3–3 cross-link in the epidemic strains as a consequence of intrinsically higher l,d-transpeptidase activity would support their elevated intrinsic resistance to β-lactam treatment.

The presence of multimers indicates the cross-linking of three or more PG polysaccharide chains. Within sacculi, muropeptides are arranged with the stem peptides protruding helically around the polysaccharide chain with potentially three (47) or four (48) muropeptides per full rotation. This allows the stem peptide to interact with muropeptides on numerous neighboring polysaccharide chains. Up to octamers have been reported for the very thick PG sacculi of Gram positive Staphylococcus aureus (49, 50). Although Gram negative sacculi are comprised of only one to three layers of PG chains (51), trimers and tetramers are often observed (25, 29, 38, 52). We identified multiple putative multimers, including putative hexamers. In addition, we were able to identify numerous multimeric muropeptides on which additional enzymatic activity has occurred (22 of the 37 multimeric muropeptides annotated). This indicates these multimeric complexes are more abundant than previously thought and likely have dynamic roles in the architecture and stability of the PG.

In our previous study, we proposed the adaptive PG represents a dynamic pool of muropeptides that allowed for flexibility in response to physiological stimuli while the lower abundance of the muropeptides would not hinder the structural stability of the PG (25). Here, we also describe an adaptive PG that was present in lower abundance than core PG but demonstrated significant fluctuations in abundance due to strain or nutrient growth condition. This adaptive PG would be generated by the activity of several hydrolases as well as modifications to either the polysaccharide or stem peptide.

Endogenous hydrolases are used by bacteria for many processes, including to relax the tight cross-linked structure for the incorporation of new PG monomers during cell growth, to recycle muropeptides, and to make room in the cell wall for the insertion of protein complexes (53, 54). There are numerous types of hydrolases, each with specificity for a different structural bond within the PG. In this study, we found evidence of three hydrolases that functioned during the formation of the adaptive PG. To generate the muropeptides observed, one of these enzymes would be an amidase, while two others would catalyze unusual endopeptidase activity.

Amidases digest the NAM-alanine bond, and most bacteria contain multiple redundant amidases that can be regulated differently based on environmental conditions or stress (55, 56). The Cpx envelope stress pathway regulates the expression of some of these amidases and has important roles in both biofilm formation and antimicrobial resistance (57, 58). The most variation in amidase activity between the conditions tested was strain specific. Interestingly, PAO1 demonstrated the highest abundance of amidase activity when grown in TSB, whereas when grown under nutrient conditions resembling CF lung infections (SCFM), it was LESB58 that demonstrated the highest abundance of amidase activity. Therefore, the strain-specific changes in amidase activity on the PG could represent intrinsic differences in regulation by the Cpx pathway. This could account in part for some of the differences in biofilm formation and antimicrobial resistance seen between the distinct strains.

With respect to the evidence for endopeptidase activity, we identified products of a d,l-endopeptidase, which digests the glutamate-*m*DAP bond in the stem peptide. d,l-Endopeptidase activity has been studied largely in Gram positive bacteria and mycobacteria, where it is important for cell division and virulence (59–61). For P. aeruginosa the best-known example of d,l-endopeptidase activity is Tse1, which is injected via the type VI secretion system into competing bacterium to degrade their PG and cause lysis (62). As Tse1 (PA1844) is a secreted protein, there should be no effect on its own PG composition. However, the P. aeruginosa amidase AmpDh3 was recently shown to be exported via the type VI secretion system (63), although it has also been shown to be periplasmically localized and functions in coordination with two homologs (64, 65). Therefore, whether Tse1 can be localized at low concentrations to the periplasm or there is another d,l-endopeptidase encoded by the P. aeruginosa genome will be of interest. In addition, one product of d,l-endopeptidase activity is the NAG-NAM attached to a two-amino-acid stem peptide, i.e., AE (Fig. 2). This product is recognized by the host immune system through the nucleotide-binding oligomerization domain-containing protein 2 (NOD2) (66, 67). The strain-specific enrichment of d,l-endopeptidase activity likely contributes to the observed differences in virulence between the distinct strains of P. aeruginosa.

The second endopeptidase that we detected evidence for was an l,d-endopeptidase. This peptidase digests between the l-chiral center of the *m*DAP and the d-alanine of the 3–4 cross-link. We cannot confirm whether the identified AE*m*AAx muropeptides were the result of l,d-endopeptidase activity or the result of an unusual addition of a 6th amino acid to AE*m*AA. However, the l,d-endopeptidase designation is more likely, as both l,d-endopeptidases that degrade the alanine-glutamate bond in Gram-positives (68, 69) and l,d-carboxypeptidases that degrade the *m*DAP-alanine bond in monomers have been identified (70, 71). The presence of the AE*m*AAx muropeptides were highly diminished when grown under the CF lung infection nutrient conditions (18- to 40-fold lower than when grown in TSB) across all the strains tested. Therefore, the decrease in l,d-endopeptidase activity potentially is important during infections for P. aeruginosa in general.

Variations to the typical stem peptide sequence, AE*m*AA, were found at the 2nd, 3rd, 4th, and 5th positions. The conversion of the 2nd position glutamate to glutamine influences cross-linking in the Gram positive Streptococcus pneumoniae (72). In this study, we identified an enrichment of 2nd position glutamine in PG from SCFM cultures as well as strain-specific enrichment. However, this did not mirror the enrichment noted for either the 3–4 or 3–3 cross-links and indicates the conversion to glutamine is not as important for cross-linking in P. aeruginosa. However, glutamate amidation was enriched under conditions like CF lung infections, indicating the glutamine in the stem peptide may have a role during infections. We also identified numerous muropeptides with unique amino acids incorporated at either the 4th or 5th position of the stem peptide. l,d-Transpeptidases and/or d,d-transpeptidases are responsible for the addition of d-amino acids to the stem peptide terminus (73). With V. cholerae, l,d-transpeptidases generate the AE*m*Ax in the PG, while the AE*m*x stems are produced using the PG precursor synthesis pathway within the cytosol prior to assembly into the PG (74). Here, we did see a differential enrichment between AE*m*x and AE*m*Ax, where AE*m*Ax predominated. Whether this represents the existence of distinct pathways in P. aeruginosa remains to be determined. In addition, stem peptides with unique terminal amino acids have been implicated in bacterium-to-bacterium competition (75) and may have a role in regulation or cell signaling (76–78). Therefore, the observed strain-specific enrichment of these terminal amino acids to the stem peptide indicate an intrinsic mechanistic difference between the epidemic and laboratory strains.

The identification of unique substitutions of the 3rd amino acid in the stem peptide is highly unusual. The presence of an *m*DAP in the 3rd position is considered a model Gram negative PG feature. However, alterations in this 3rd position in Gram negative bacteria are possible. For instance, Gram negative Thermus thermophilus PG contains a unique l-ornithine in the 3rd position of its stem peptides (79, 80), and the PG of several species of *Porphyromonas* (previously *Bacteroides*) contains either lysine or *m*DAP (81). Within Escherichia coli, the enzyme responsible for adding the 3rd amino acid to the stem peptide, MurE, can incorporate unique diamino acids *in vitro*, including lanthionine, cystathionine, and d-lysine, albeit at lower turnover rates than *m*DAP (82, 83). However, this study provides the first evidence of PG stem peptides with an altered 3rd position amino acid present within the mature sacculi of P. aeruginosa. The *m*DAP stimulates innate immune responses in mammalian cells (84). Therefore, the ability to reduce such constituents during infection would provide an advantage. The fact that the 3rd position substitution was enriched when grown in SCFM that has a nutrient composition similar to that found in CF lung infections is interesting. In our previous study, we identified two putative muropeptides that can now be annotated as AEKA-A*m*EA and AEEA-A*m*EA and were enriched in P. aeruginosa when growing within a biofilm (25). Biofilms are linked to chronic infections (85–87) and enhanced antimicrobial resistance (88). Therefore, the presence of these muropeptides with 3rd position substitution within the PG is likely advantageous during infections.

The remaining modifications to the PG we identified as part of the adaptive PG involved de-N-acetylation of the polysaccharide as well as the previously undocumented *m*DAP deamination or O-methylation of the stem peptide. The deN modification removes the acetyl group from either the NAG or the NAM of the polysaccharide backbone. Removal of the acetyl group impedes the binding of host-produced lysozyme, thereby restricting digestion of the PG backbone during host immune defense (89, 90). Here, we demonstrated an increase of NAG deN muropeptides within the epidemic strains compared to PAO1 and PA14. Therefore, the deN PG backbone increases lysozyme resistance and thus participates in the heightened ability of the epidemic strains to evade host immune defenses and hence cause significant bacterial infections.

The putative PG modifications, deA and O-methylation, have not been documented before in the mature PG sacculi of P. aeruginosa. Methylation on either the 2nd position glutamate, *m*DAP, or the terminal alanine has been documented in Mycobacterium smegmatis PG (91); however, the biological function of this modification is unknown. Bacterial outer membrane proteins are known to be posttranslationally methylated, and these modifications are important for virulence (92, 93). The increase of the O-methylated stem peptides in SCFM (3.11- to 6.86-fold) indicates it has a role during P. aeruginosa infection. The deA of the *m*DAP residue has not been documented previously; however, amidation of the *m*DAP has been identified (94–96), where in some bacterial species amidated *m*DAP predominates (97, 98). The presence of amidated *m*DAP within the mature PG of some bacterial strains has been shown to stabilize the cell wall, thus increasing resistance to antimicrobials and lysozyme (95, 99). In addition, the amidation of *m*DAP can alter the recognition of these muropeptides by the NOD proteins, thereby moderating host immune detection (96, 100). It is possible that the deA of the *m*DAP residues within PG play roles similar to amidation. In addition, deA of PG muropeptides was found mostly in conjunction with amidase activity and, therefore, may also have a role in cell division.

The impact of the nutrient conditions of the growth medium is known to significantly influence the metabolic function and gene expression of P. aeruginosa (30, 101–103). This work highlights this to show that the overall enzymatic activity functioning on the peptidoglycan is also affected by nutrient conditions. Future studies will need to account for the nutrient content of the growth medium to elucidate the enzymatic activity that produces specific PG modifications to link peptidoglycomics to proteome/transcriptome analyses. Understanding the enzymatic activity that functions on PG has historically been difficult to study. Genomic analyses indicate the clinical strains of P. aeruginosa can differ in single nucleotide polymorphisms (SNPs) within PG-modifying enzymes, including transpeptidases, transglycosylases, carboxypeptidases, lytic transglycosylases, and amidases (7, 13). These SNPs may account for some of the noted strain-to-strain variations observed in this study. However, many genes involved in PG synthesis and modification have redundant and/or overlapping activities (65, 104). Therefore, disruption of a single gene may not eliminate or significantly vary the modifications observed on the PG. To complicate matters further, the regulation of genes responsible for PG modification through distinct sensory systems could also be responsible for the variation between strains and growth conditions. For example, the regulatory two-component system (TCS) BfmRS has been shown to regulate many genes involved in PG synthesis and modification in Acinetobacter baumannii (105) and has been shown to differ in activity between P. aeruginosa clinical strains (106). Another TCS, the stress response CpxRA system, can regulate lytic transglycosylase and l,d-transpeptidase expression in E. coli (107, 108). Therefore, understanding the enzymatic activities responsible for the PG modifications identified will require more detailed analyses.

This work delivers a robust and reproducible workflow for accurately determining PG composition between samples that can be used to assess global PG fluctuations in response to changing growth conditions or external stimuli. Our approach represents a significant advance toward a consistent and reproducible analysis of PG structure with clinical applications and puts peptidoglycomics on an equal footing with other omics disciplines to study this critical bacterial biopolymer.

## MATERIALS AND METHODS

### Bacterial culture.

Four distinct isolates of P. aeruginosa were studied. Two are the well-studied laboratory strains PAO1 and PA14. In addition, two isolates of the epidemic strains were chosen. LESB58 is an isolate of the Liverpool epidemic strains isolated from a patient in the United Kingdom in 1995 (3), whereas LESlike1 was isolated from a patient in Canada in 2005 (4). To examine the impact of nutritional conditions on PG composition, two growth media were also chosen, tryptic soy broth (Fisher) and synthetic CF sputum medium (SCFM), which was prepared as described in Palmer et al. (30), except individual amino acids were replaced with 5 g/liter Casamino Acids (Fisher).

For each strain and medium combination, starter cultures of 100 mL were inoculated and incubated overnight, with shaking at 200 rpm at 37°C. Cultures were diluted to an optical density at 600 nm (OD_600_) of 0.05 in four flasks each containing 1 liter of appropriate medium and incubated at 200 rpm 37°C until an OD_600_ of 0.5 was reached. Samples of each flask were measured for OD_600_ and number of CFU/mL (see Table S1 in the supplemental material). Cultures were immediately cooled on ice and pelleted at 7,000 × *g* for 10 min at 4°C. Pellets were frozen at −20°C until ready for sacculus purification. Cultures were produced in biological triplicate.

### Purification of peptidoglycan sacculi.

PG sacculi were extracted from all other cellular components by following the methodology described in Anderson et al. (25, 26). Briefly, cultures were boiled for 3 h in 20 mM sodium phosphate buffer, pH 6.5, containing 4% sodium dodecyl sulfate (SDS). SDS was removed from the extracted sacculi by repeated washes in 20 mM sodium phosphate buffer, pH 6.5, with ultracentrifugation at 70,000 × *g* for 40 min. Contaminants were digested using DNase, RNase, and amylase, followed by digestion with pronase. Final purification was accomplished by boiling in 20 mM sodium phosphate buffer, pH 6.5, containing 2% SDS for 1 h. SDS was again removed by repeated washes with H_2_O and ultracentrifugation at 70,000 × *g* for 40 min. Sacculi were lyophilized and resuspended to 10 mg/mL in H_2_O.

### Quantification of peptidoglycan.

Purified sacculi were quantified using *m*DAP concentrations similar to Torrens et al. (44, 109, 110). Resuspended sacculi were acid hydrolyzed with 6 M hydrochloric acid at 100°C for 2 h in a vacuum-sealed glass ampule. Ampules were opened and the samples were dried by heating at 100°C under vacuum over solid NaOH to neutralize the acid. Dried hydrolyzed PG was resuspended in H_2_O to double the original volume. Samples were treated with 25 mg/mL ninhydrin in 0.24 M phosphoric acid with 10.4 M acetic acid and heated at 100°C for 5 min. Samples were immediately read at *A*_434_ and compared to a standard curve produced using 2,6-diaminopimelic acid (*m*DAP) (Sigma). Each PG biological triplicate sample was tested in duplicate with technical duplication.

### Mass spectrometry of peptidoglycan.

Preparation of sacculi for mass spectrometry was the same as that in Anderson et al. (25). Briefly, resuspended sacculi were digested into individual muropeptides by digestion with 100 μg/mL mutanolysin overnight at 37°C. Muropeptides were reduced by incubation with a 1:1 volume of ~10 mg/mL sodium borohydride at room temperature for 20 min. The pH of the sample was adjusted to <4 using phosphoric acid and filtered using Nanasep MF 0.2-μm microcentrifuge filters (PALL [Canada] Ltd., Mississauga, ON, Canada).

Liquid chromatography-mass spectrometry analysis was performed at the Mass Spectrometry Facility of the Advanced Analysis Centre, University of Guelph, on an Agilent 1200 HPLC liquid chromatograph interfaced with an Agilent UHD 6540 Q-TOF mass spectrometer. Separation was achieved using a C_18_ column (50 mm by 2.1 mm, 2.7 μm; Agilent AdvanceBio peptide map) with parameters similar to Anderson et al. (25, 26). All biological replicates were analyzed in technical triplicate.

### Muropeptide structure library.

The MassHunter Personal Compound Database and Library (PCDL) v.B.07.00 (Agilent Technologies Inc., Santa Clara, CA, USA) produced in Anderson et al. (25) was further expanded to include additional PG modifications and unique structures. Unique structures were produced using ChemDraw Prime v.16.0.1.4 (Perkin-Elmer Inc., Waltham, MA, USA). The PG modifications and combinations were predicted using Microsoft Excel and uploaded into the PCDL library. In total >170,000 different muropeptide structures were included (Table S2).

### Bioinformatic analysis.

Using the Masshunter Profinder v.B08.00 (Agilent Technologies Inc., Santa Clara, CA) molecular feature extraction algorithm, mass spectra were processed similarly to Anderson et al. (25). Initial *m/z* detection thresholds were set to 350 counts with an extraction window of 30 ppm. The compound threshold was set to an absolute height of 550 counts in two-thirds of samples in at least one group. Consensus muropeptides were used for recursive feature detection using the find-by-ion algorithm with a 40-ppm extraction window. Aligned compounds were imported into Mass Profiler Professional (MPP) v.14.9.1 (Agilent Technologies, Inc., Santa Clara, CA) for differential analysis. Compounds representing in-source loss of NAG were merged. The data were normalized using the *m*DAP quantification values and baselined to the median intensity of each muropeptide across samples. Identification of muropeptides was conducted using MPP IDBrowser Identification combined with the muropeptide (PCDL) library (Table S2). Muropeptide annotations were manually confirmed using the MS/MS spectra. Unique muropeptides were exported for a preferred MS/MS fragmentation to confirm identity. Strain-to-strain and strain-to-medium compound abundance fluctuations were assessed for significance using a moderated *t* test and corrected using Benjamini-Hochberg false discovery rate (FDR) (31). Data were grouped using unsupervised hierarchical clustering using normalized intensity values with Euclidean similarity measure and Wards linkage rule. To assess global variations of each PG characteristic, the normalized intensity values of all muropeptides determined in MPP were uploaded into Perseus v.1.6.2.2 (111) for 1D annotation calculations (32). Each muropeptide was assigned to various 1D annotation categories based on the structural characteristics and assessed for significance (*P*  < 0.05, FDR < 0.05, s0 = 1). Prism 5.0f (GraphPad Software Inc., San Diego, CA) was used to produce all graphs.

### Data availability.

Raw MS and MS/MS spectra are deposited in the publicly accessible repository figshare at https://doi.org/10.6084/m9.figshare.19119110.v1.
