# Supplementary figures and images for: Pseudomonas aeruginosa Alters Peptidoglycan Composition under Nutrient Conditions Resembling Cystic Fibrosis Lung Infections
Source: mSystems. 2022 May 12;7(3):e00156-22. doi: 10.1128/msystems.00156-22 (PMC9239049; doi:10.1128/msystems.00156-22)

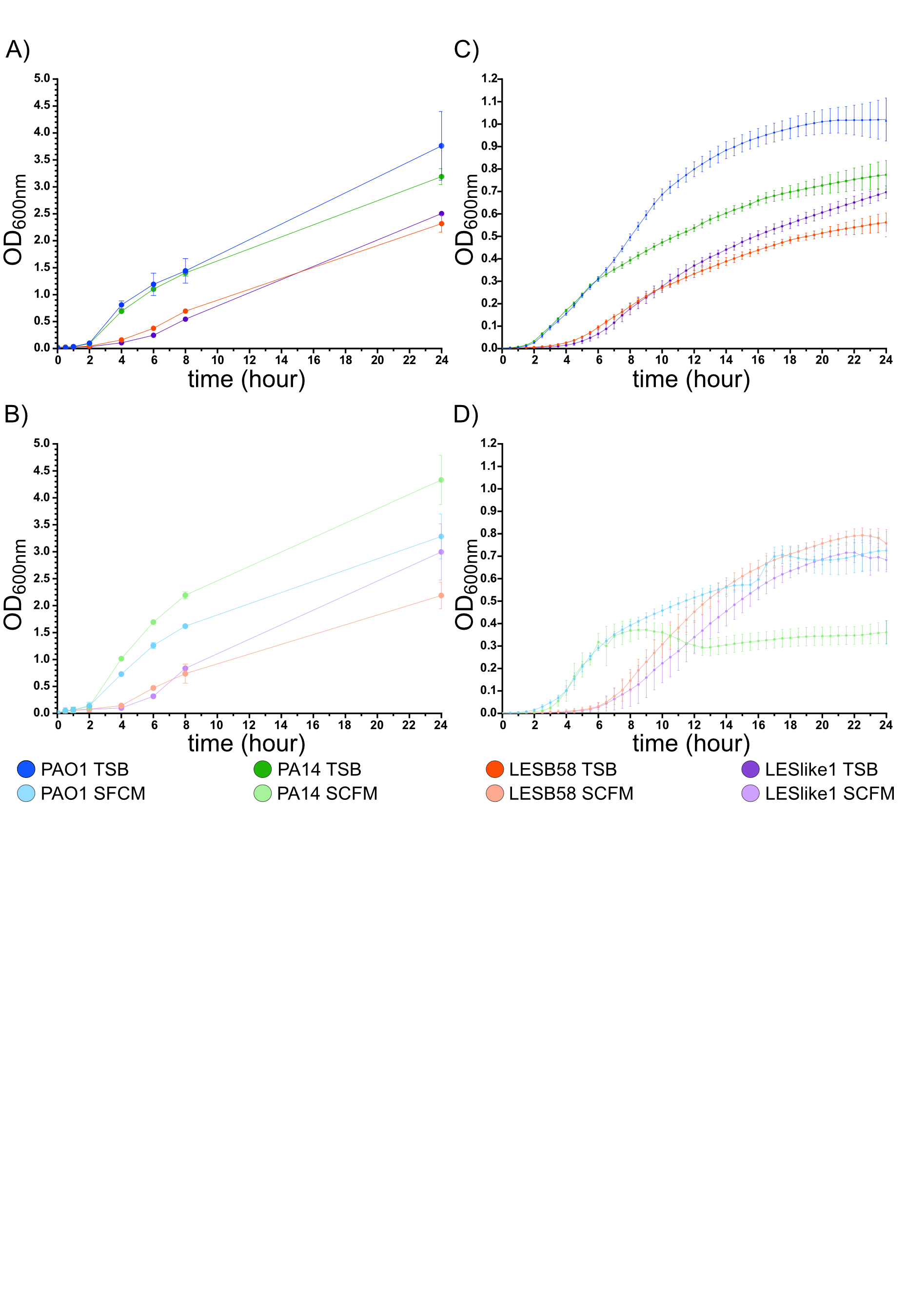

Supplement: FIG S1 [file msystems.00156-22-s0001.tif]

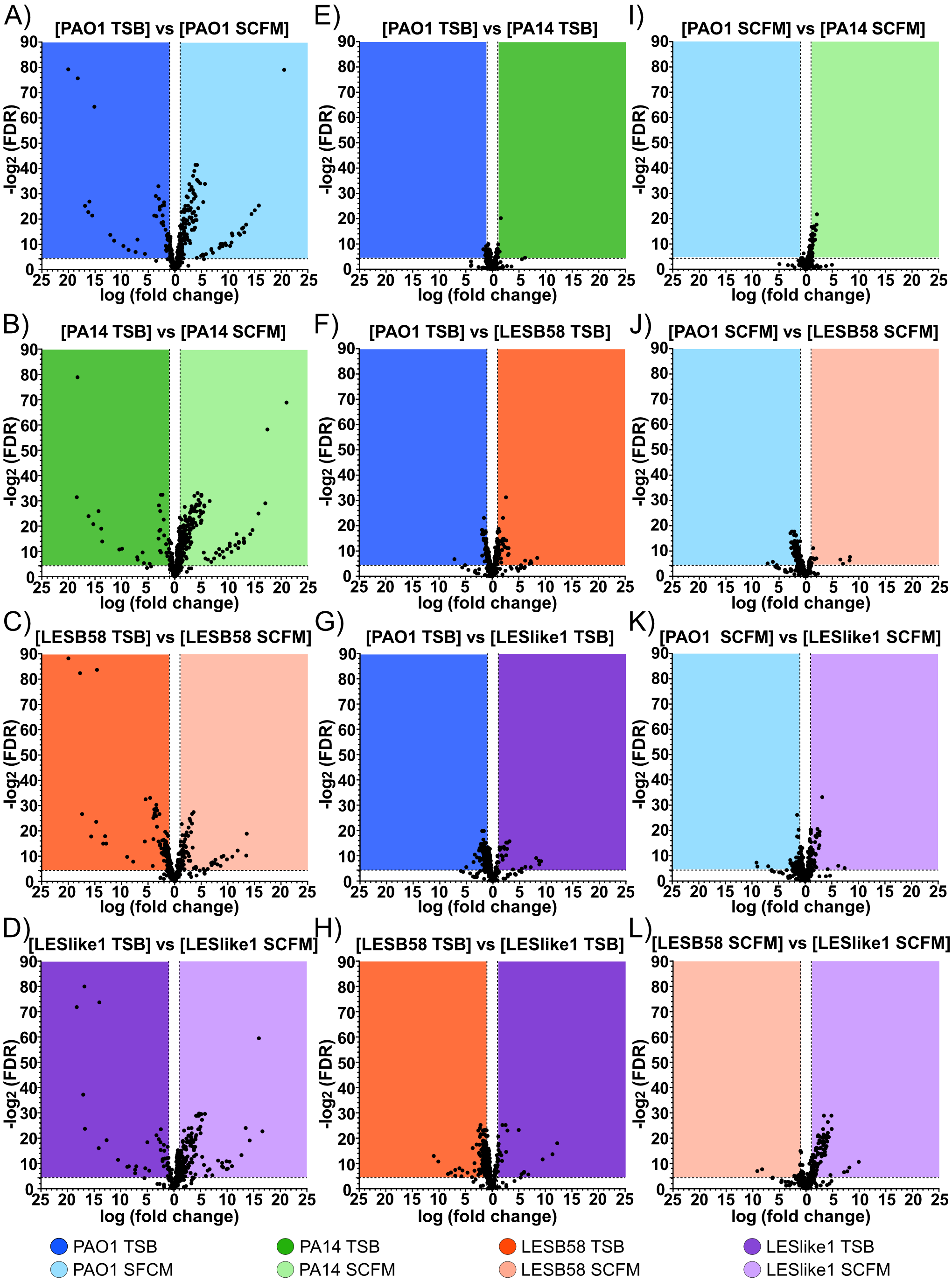

Supplement: FIG S3 [file msystems.00156-22-s0003.tif]

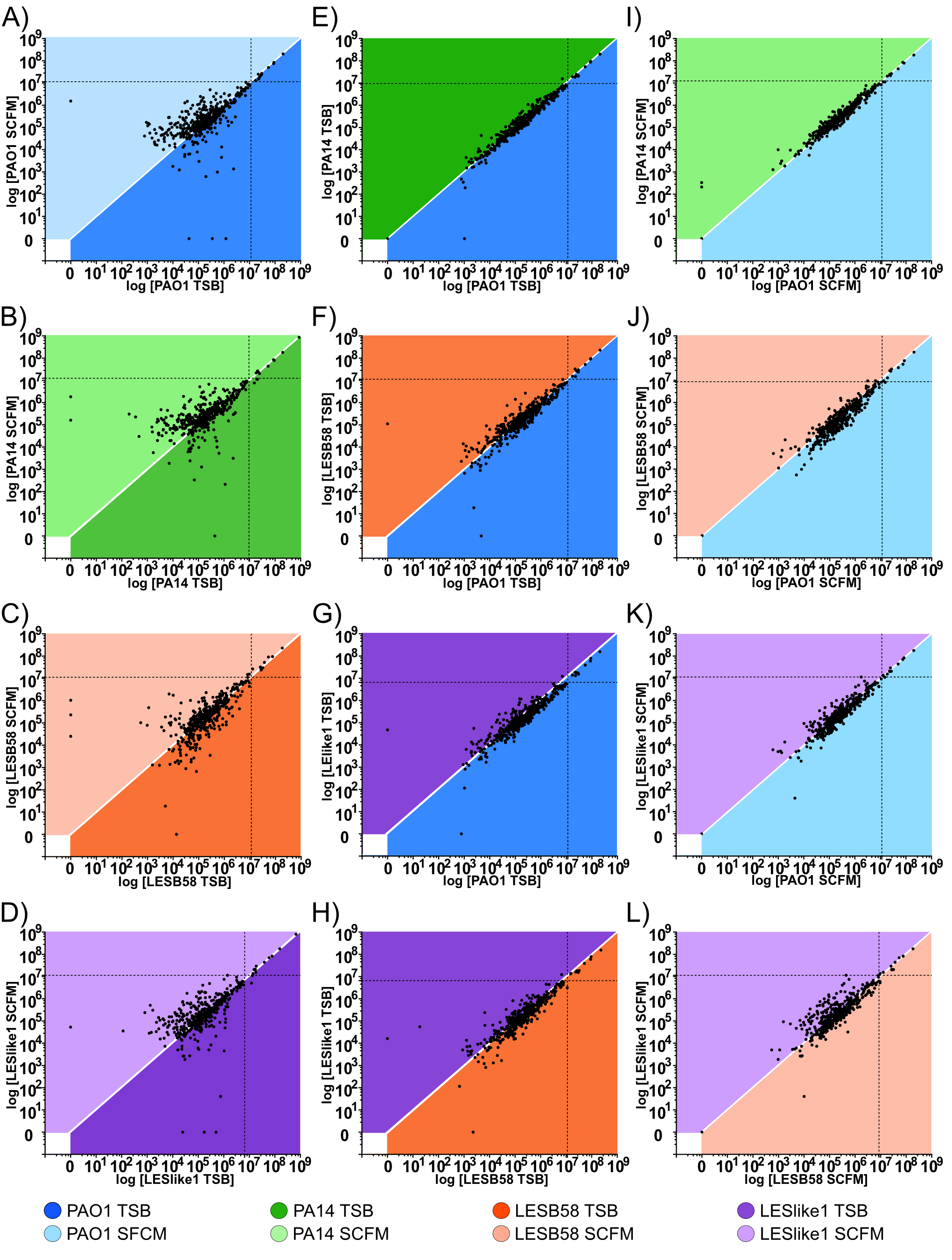

Supplement: FIG S2 [file msystems.00156-22-s0002.tif]
