# Supplementary material for: Pseudomonas aeruginosa Alters Peptidoglycan Composition under Nutrient Conditions Resembling Cystic Fibrosis Lung Infections
Source: mSystems. 2022 May 12;7(3):e00156-22. doi: 10.1128/msystems.00156-22 (PMC9239049; doi:10.1128/msystems.00156-22)

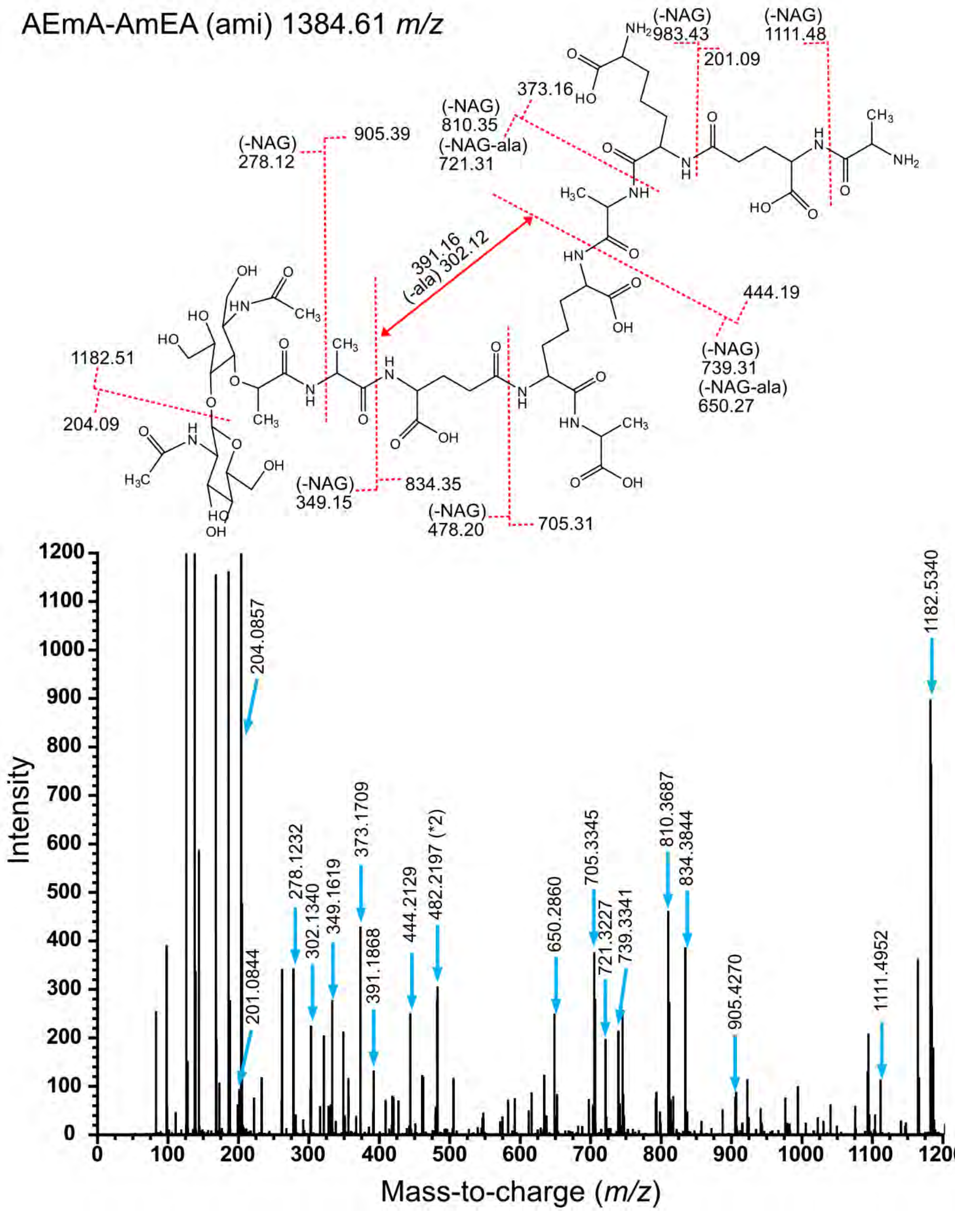

AEmA-AmEA (ami) 1384.61 m/z
(-NAG)
983.43
(-NAG)
1111.48
201.09
373.16
(-NAG)
810.35
(-NAG-ala)
721.31
(-NAG)
278.12
905.39
391.16
(-ala) 302.12
444.19
(-NAG)
739.31
(-NAG-ala)
650.27
1182.51
204.09
(-NAG)
349.15
834.35
(-NAG)
478.20
705.31
Intensity
Mass-to-charge (m/z)
204.0857
201.0844
278.1232
302.1340
349.1619
373.1709
391.1868
444.2129
482.2197 (*2)
650.2860
705.3345
721.3227
739.3341
810.3687
834.3844
905.4270
1111.4952
1182.5340

2x(NAG-NAM) - 976.40 *m/z*

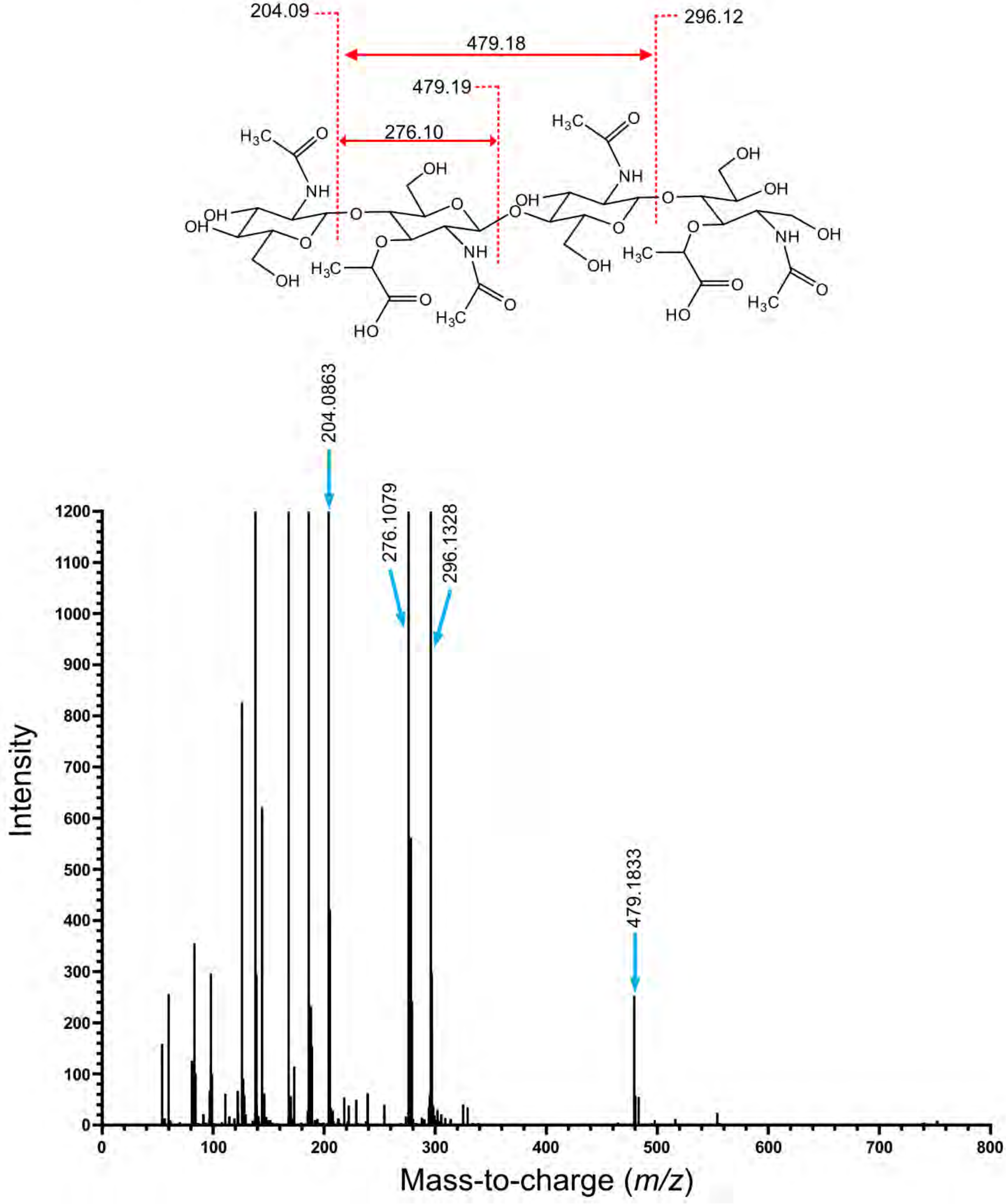

AEmAmA - 1184.53 *m/z*

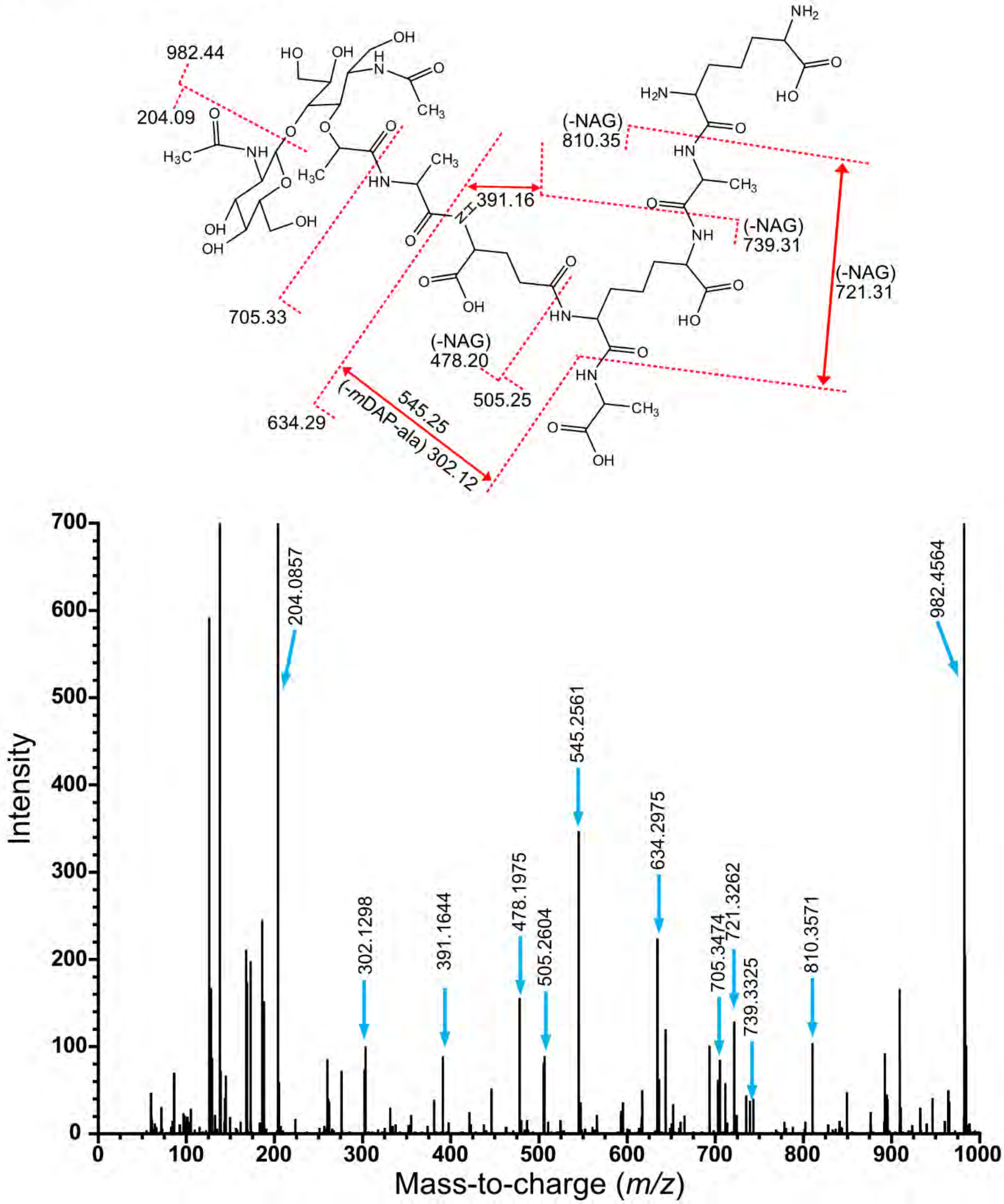

AEmA-mA-mEA - 2036.89 *m/z*

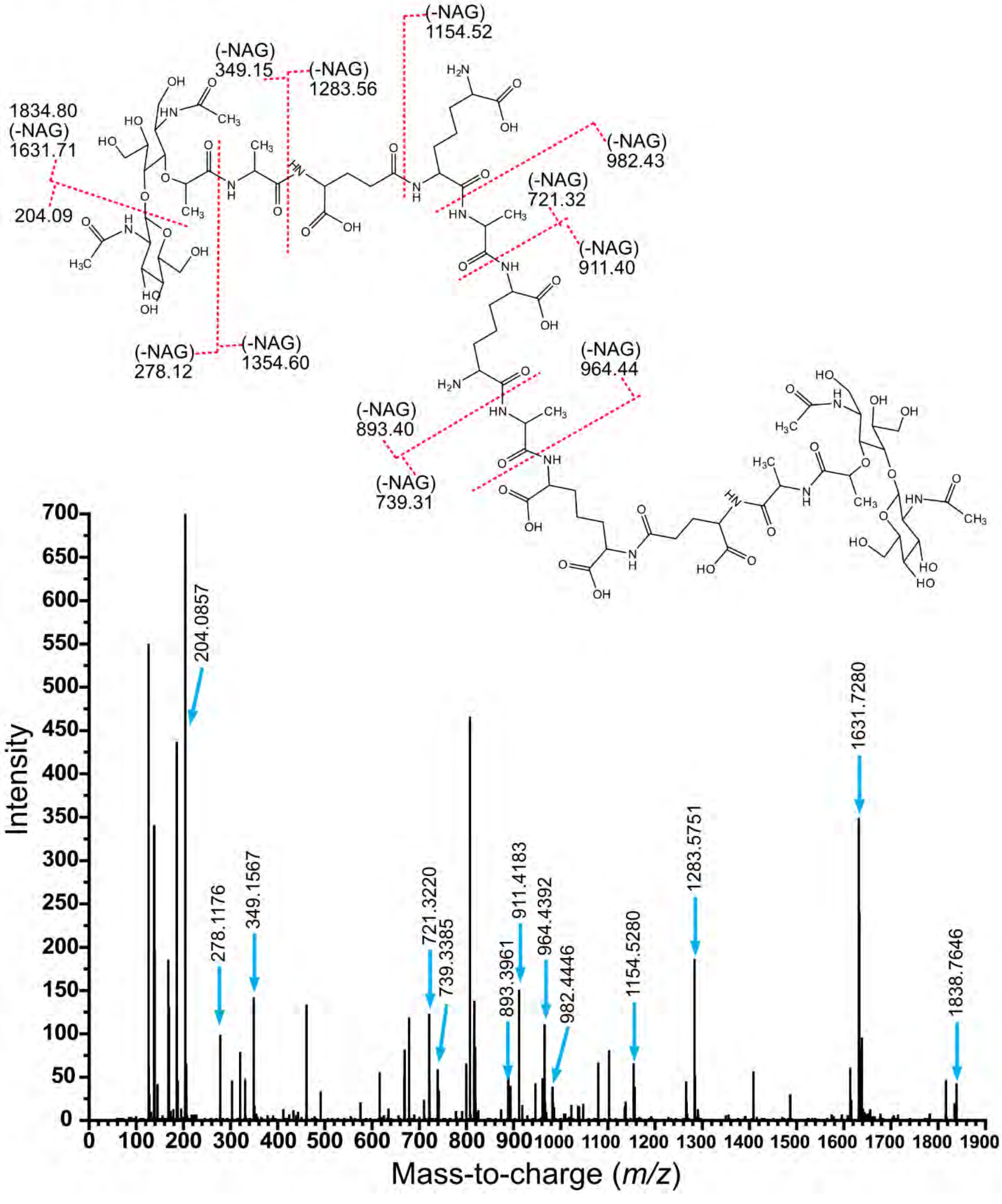

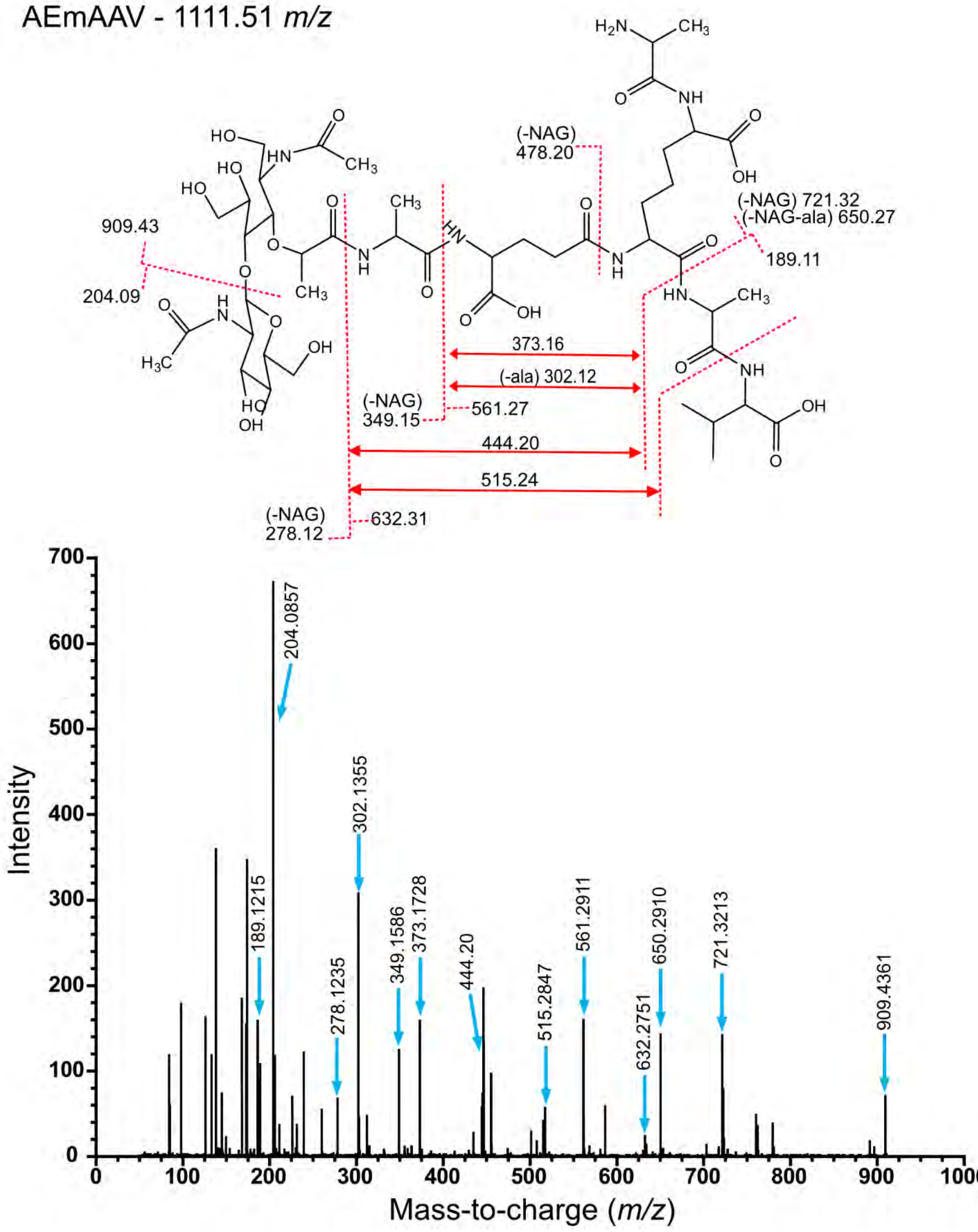
AEmAAV - 1111.51 m/z
909.43
204.09
(-NAG)
478.20
(-NAG) 721.32
(-NAG-ala) 650.27
189.11
373.16
(-ala) 302.12
(-NAG)
349.15
561.27
444.20
515.24
(-NAG)
278.12
632.31
Intensity
Mass-to-charge (m/z)
204.0857
302.1355
189.1215
278.1235
349.1586
373.1728
444.20
515.2847
561.2911
632.2751
650.2910
721.3213
909.4361

AEmAAS - 1099.48 *m/z*

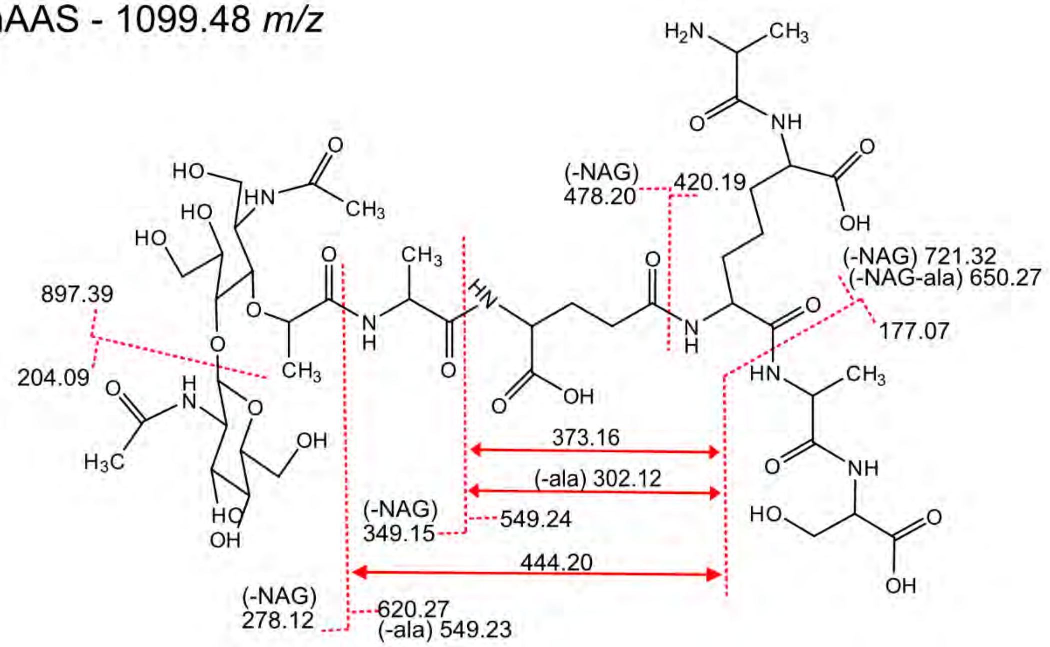

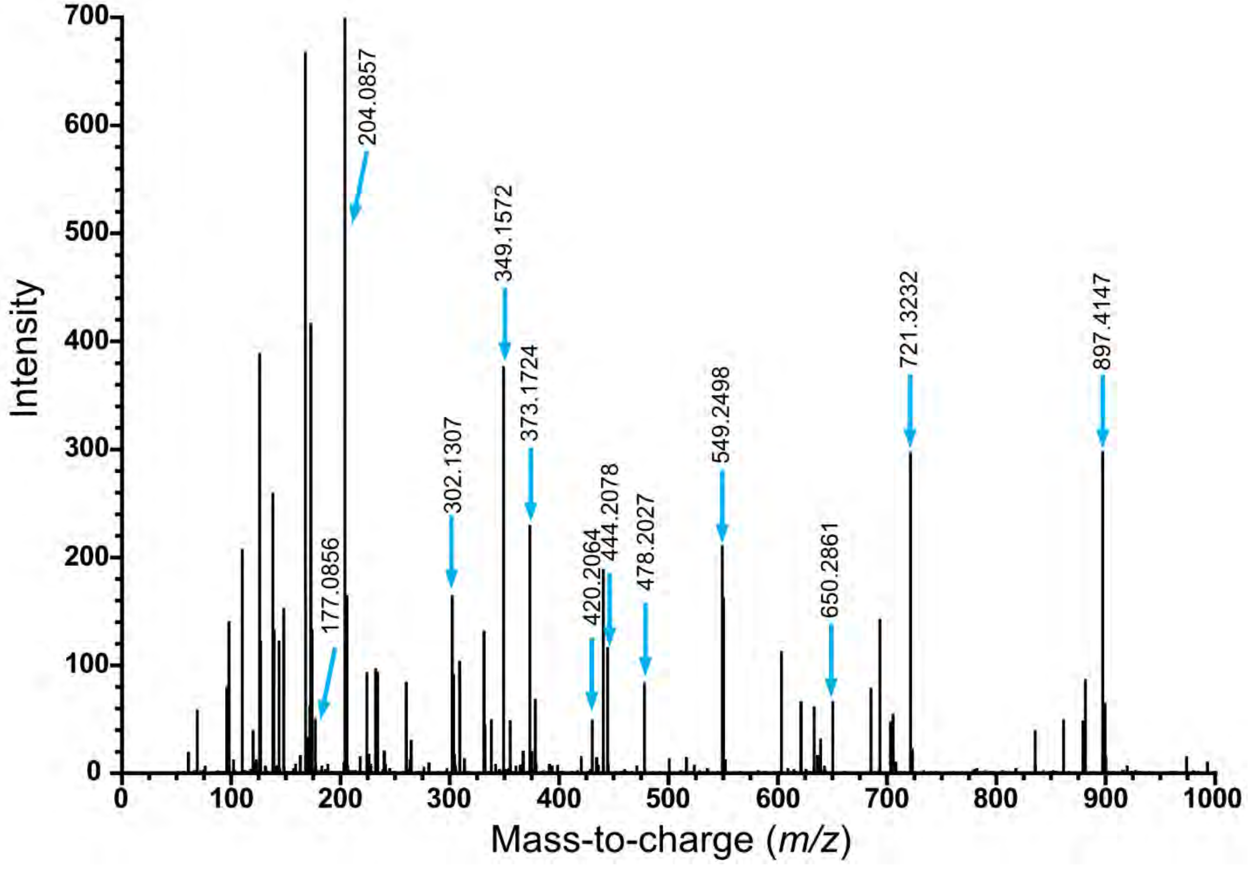

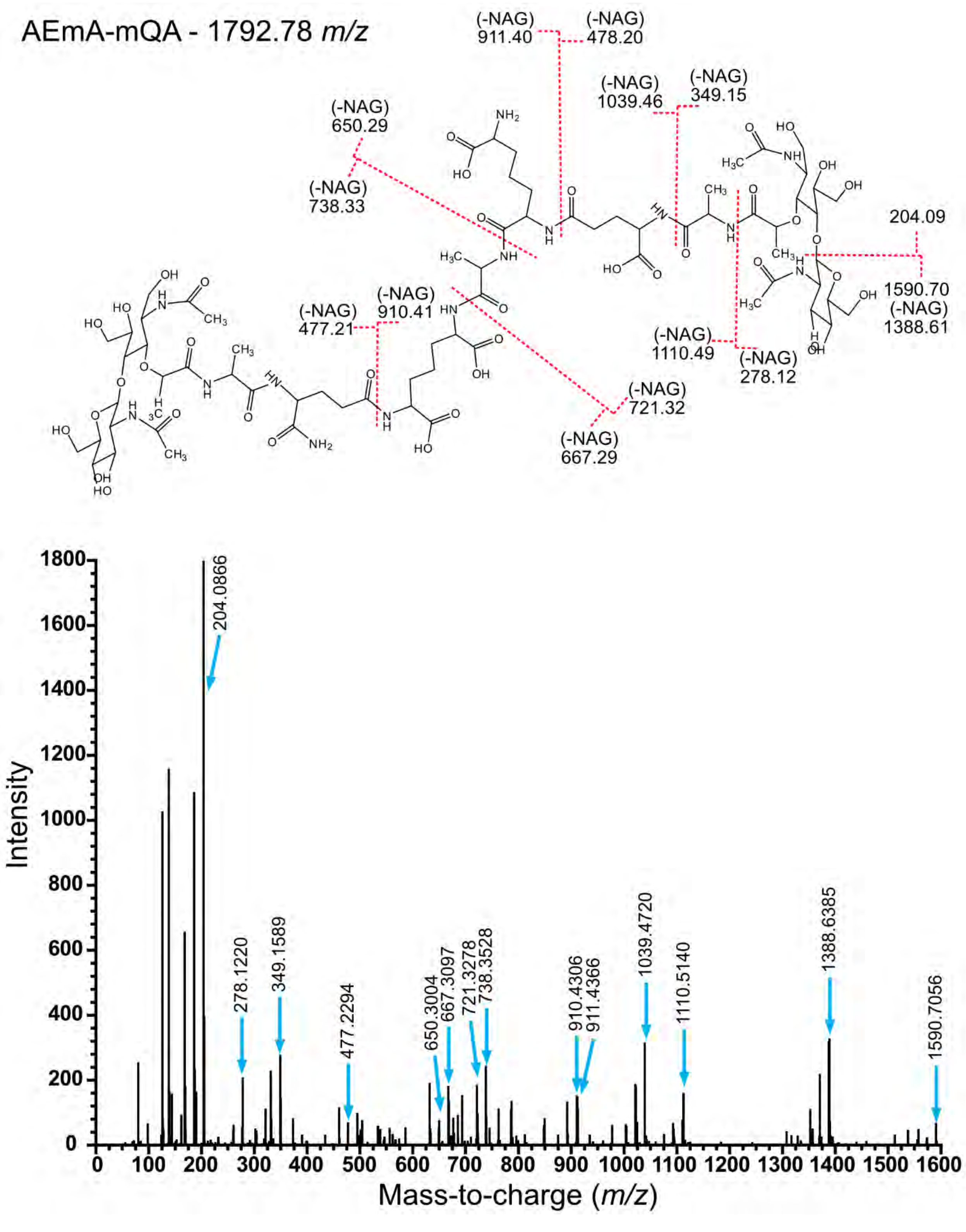

AEmA-mQA - 1792.78 m/z
(-NAG) 911.40
(-NAG) 478.20
(-NAG) 1039.46
(-NAG) 349.15
(-NAG) 650.29
(-NAG) 738.33
204.09
1590.70
(-NAG) 1388.61
(-NAG) 477.21
(-NAG) 910.41
(-NAG) 1110.49
(-NAG) 278.12
(-NAG) 721.32
(-NAG) 667.29
Intensity
Mass-to-charge (m/z)
204.0866
278.1220
349.1589
477.2294
650.3004
667.3097
721.3278
738.3528
910.4306
911.4366
1039.4720
1110.5140
1388.6385
1590.7056

AEmG - 927.39 *m/z*

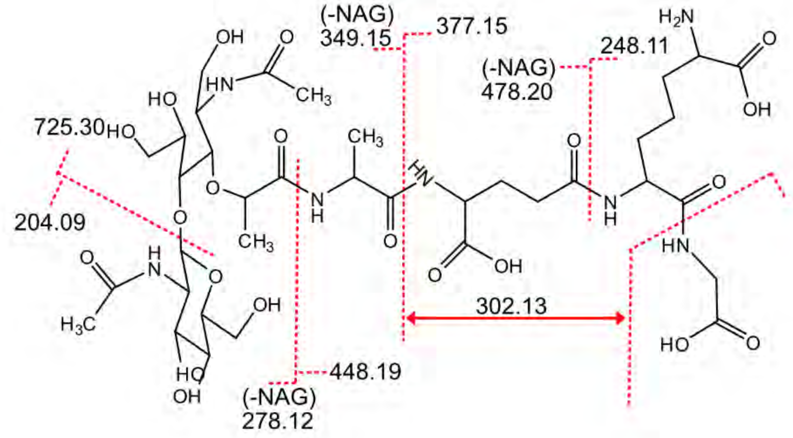

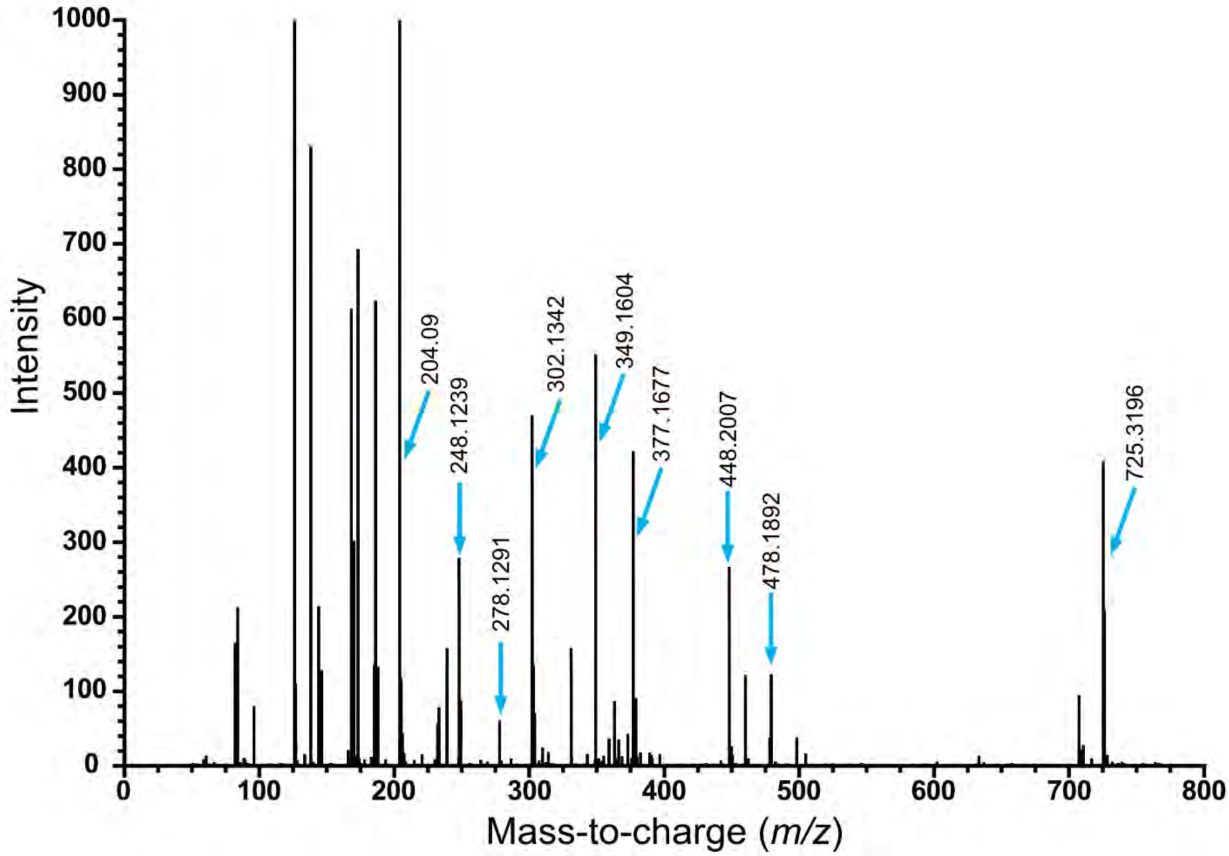

AEmAV - 1040.48 *m/z*

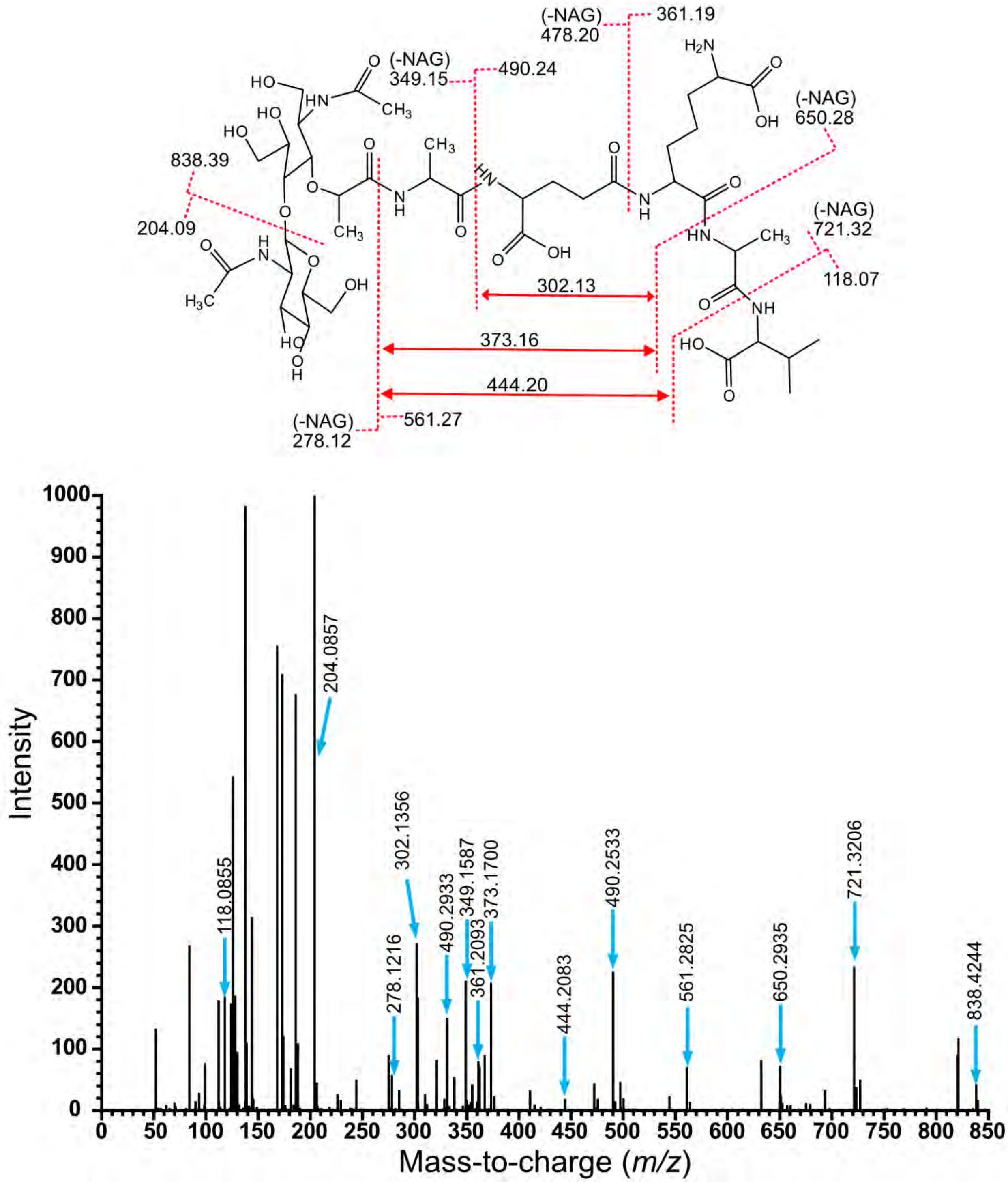

AEKA - 897.42 *m/z*

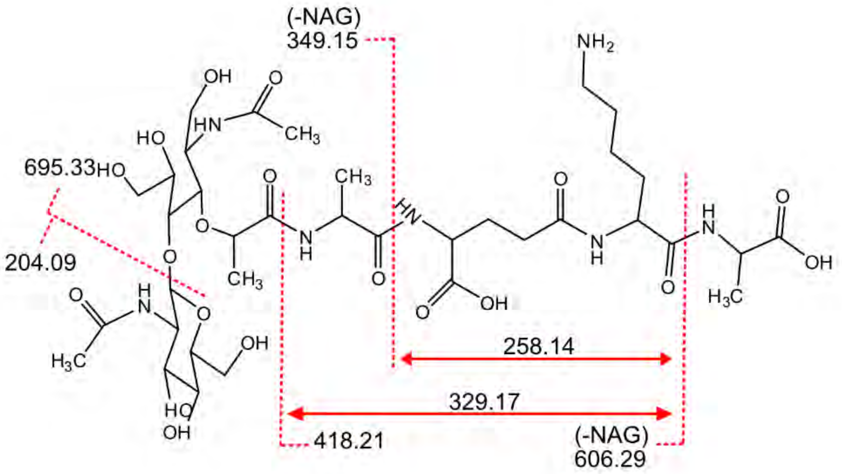

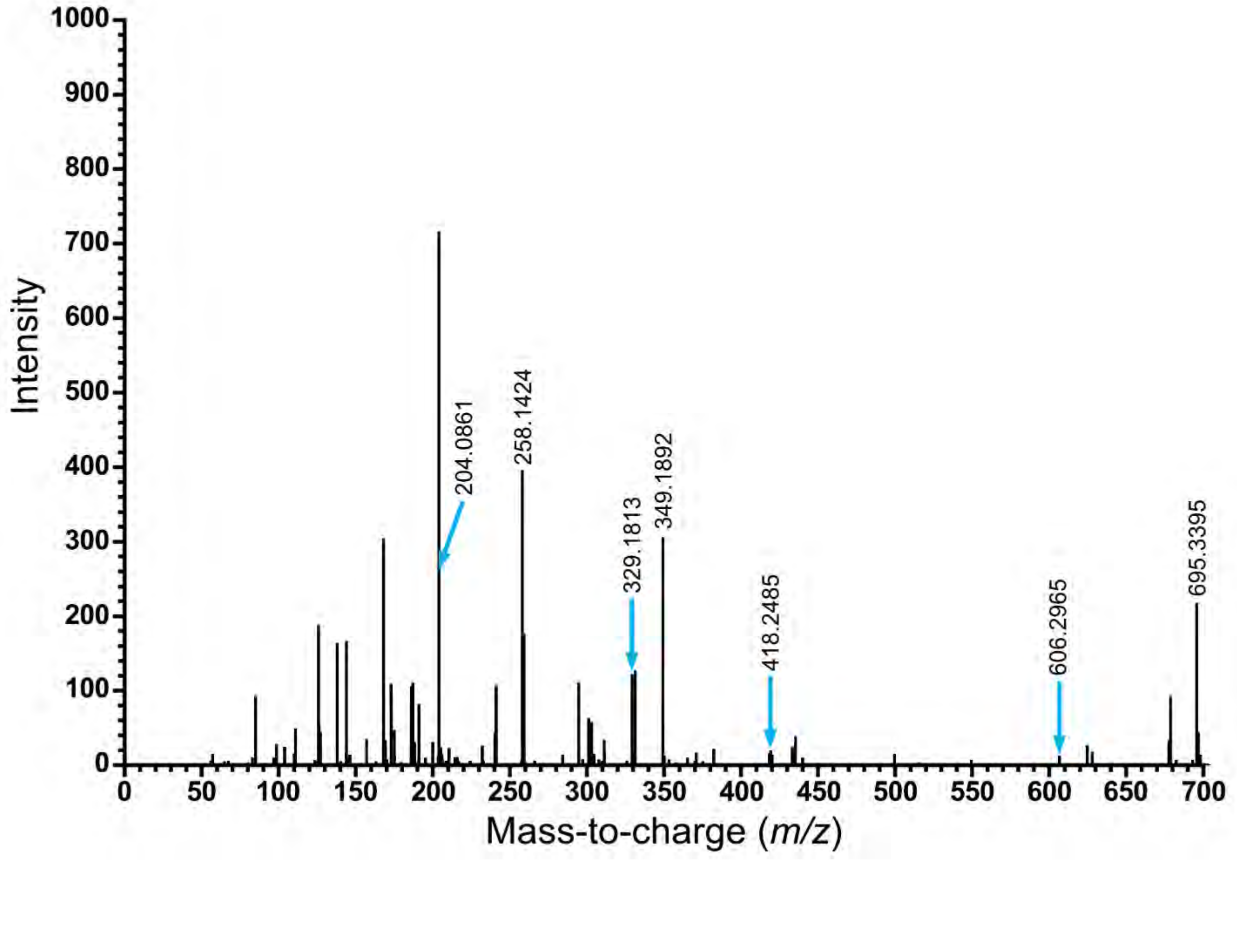

AEVA - 868.39 *m/z*

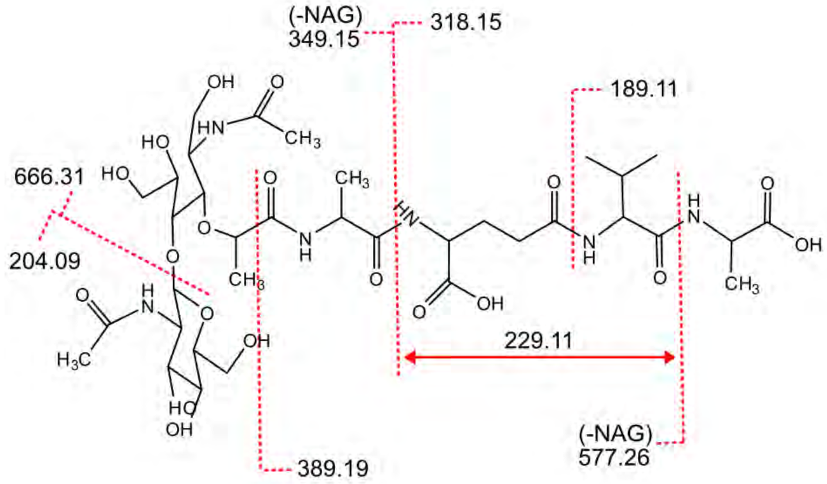

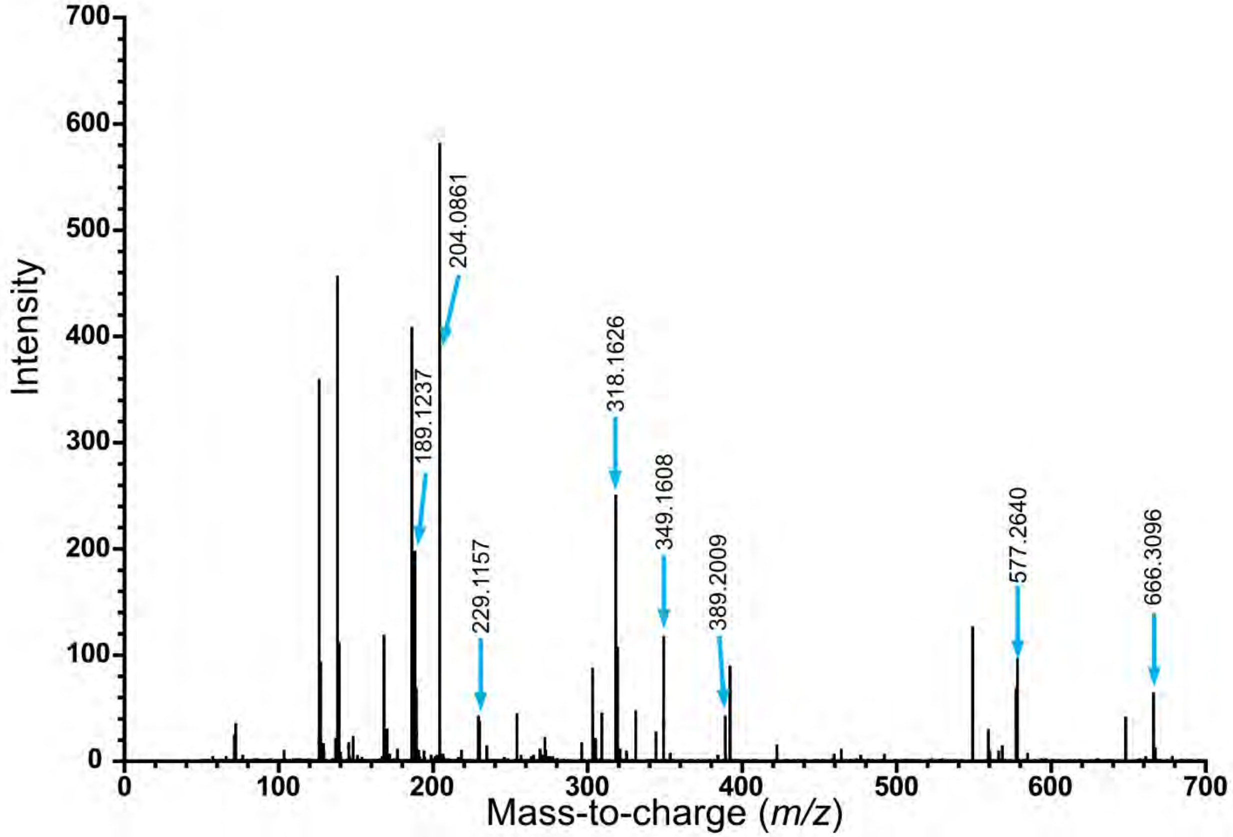

AEKA-AmEA - 1820.83 *m/z*
retention time 15.18 min

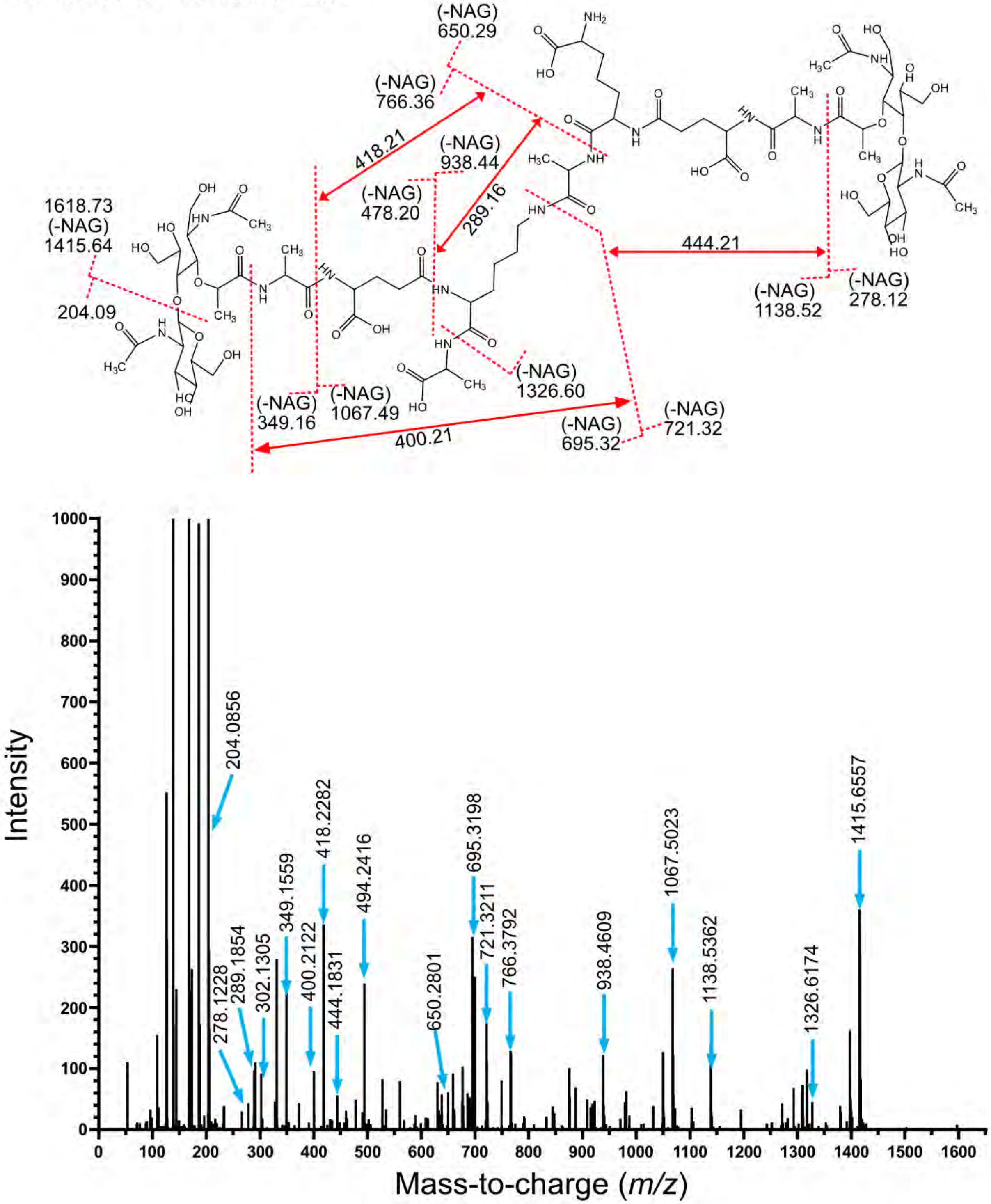

AEmA (meth) - 955.42 *m/z*

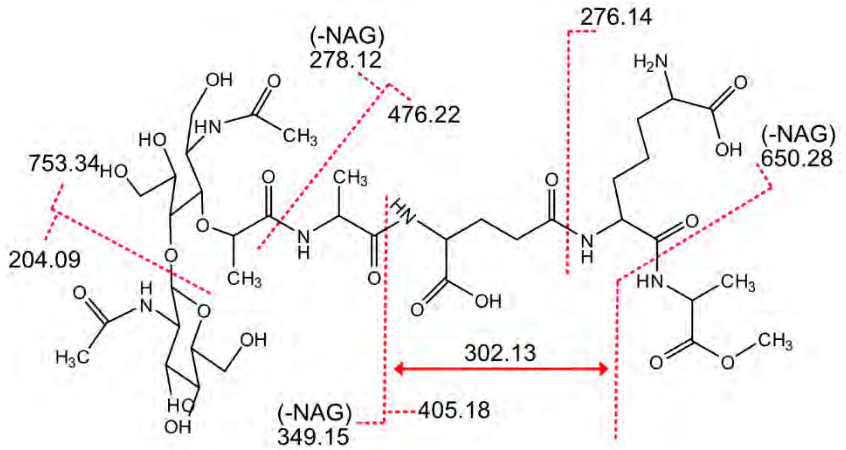

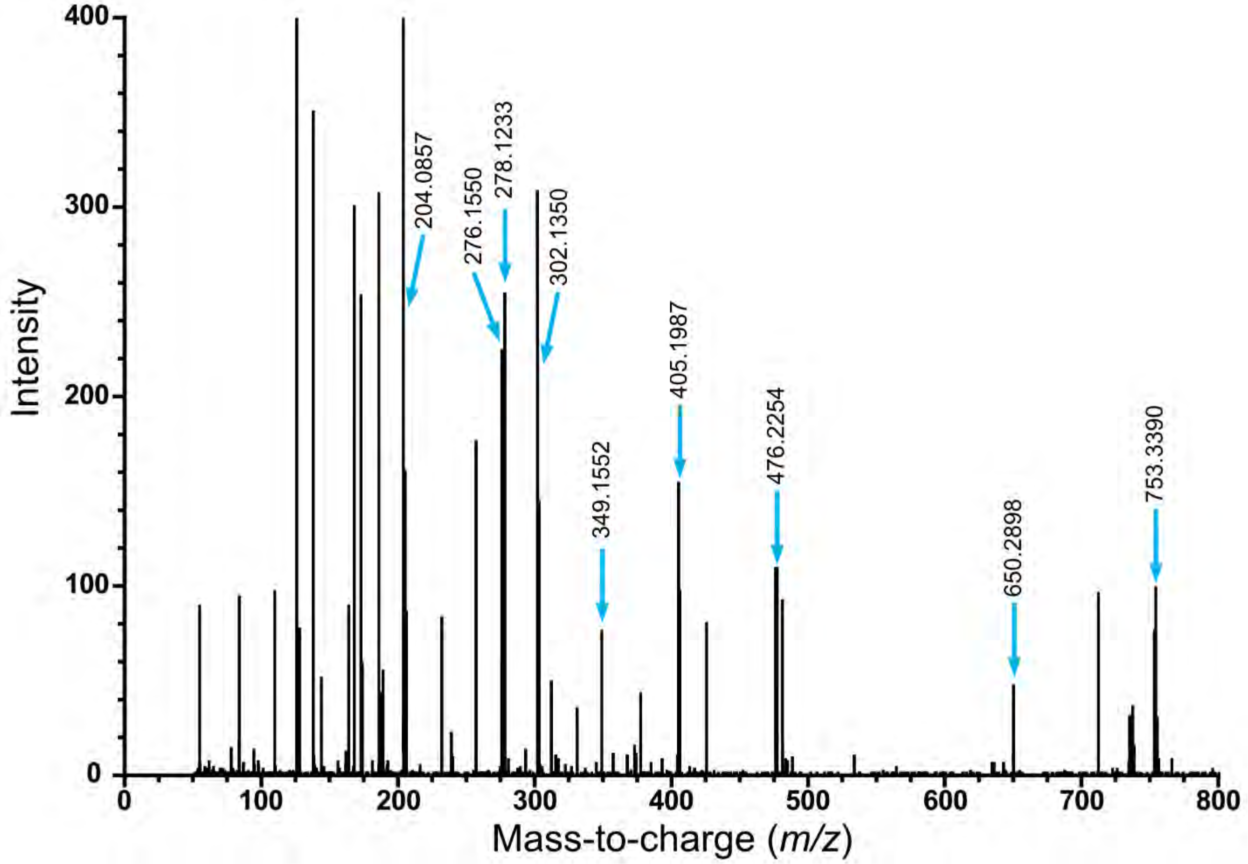

AEmAA (deA) - 1013.42 *m/z*

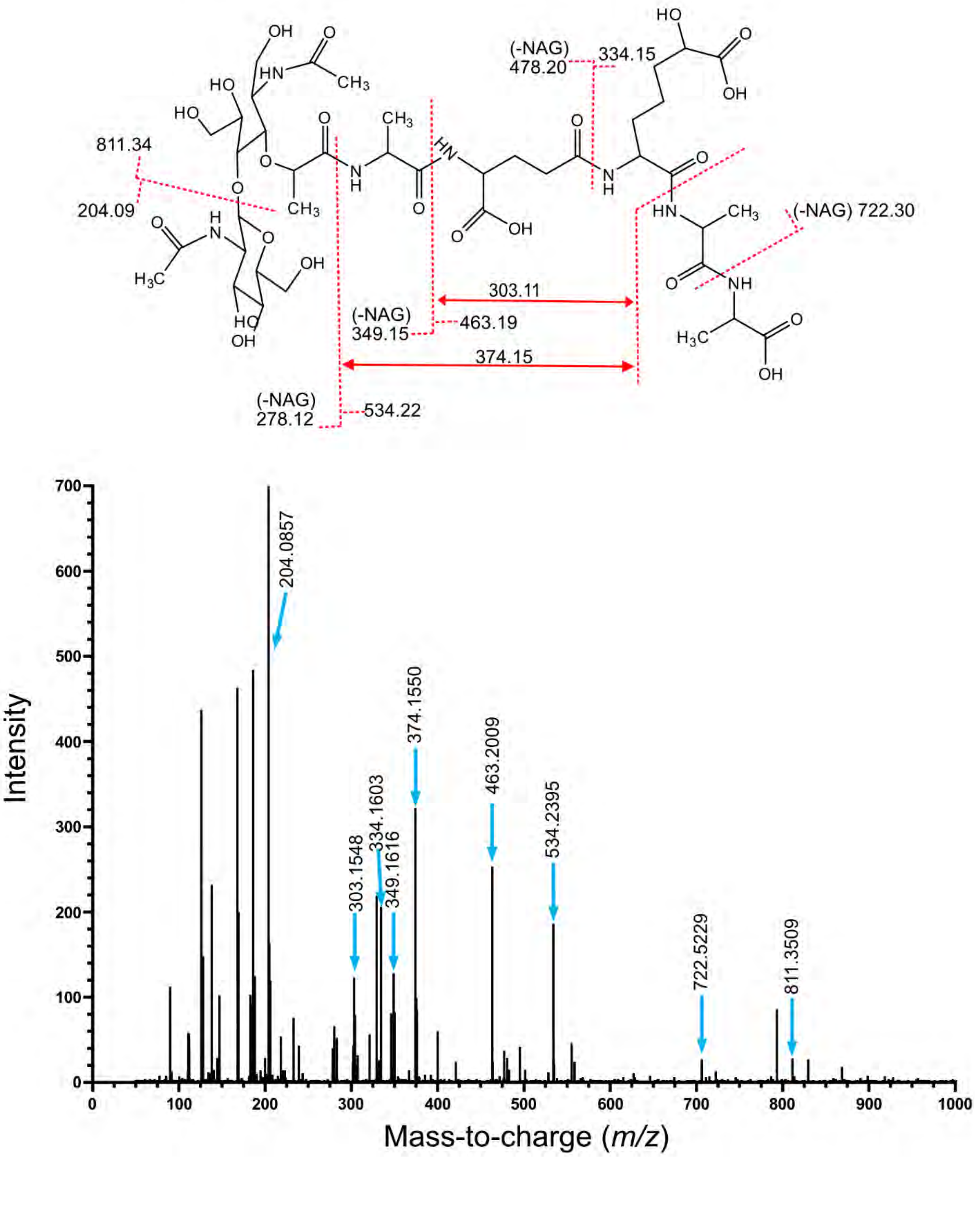

AEmA-AmEA (ami, deA) - 1385.61 *m/z*

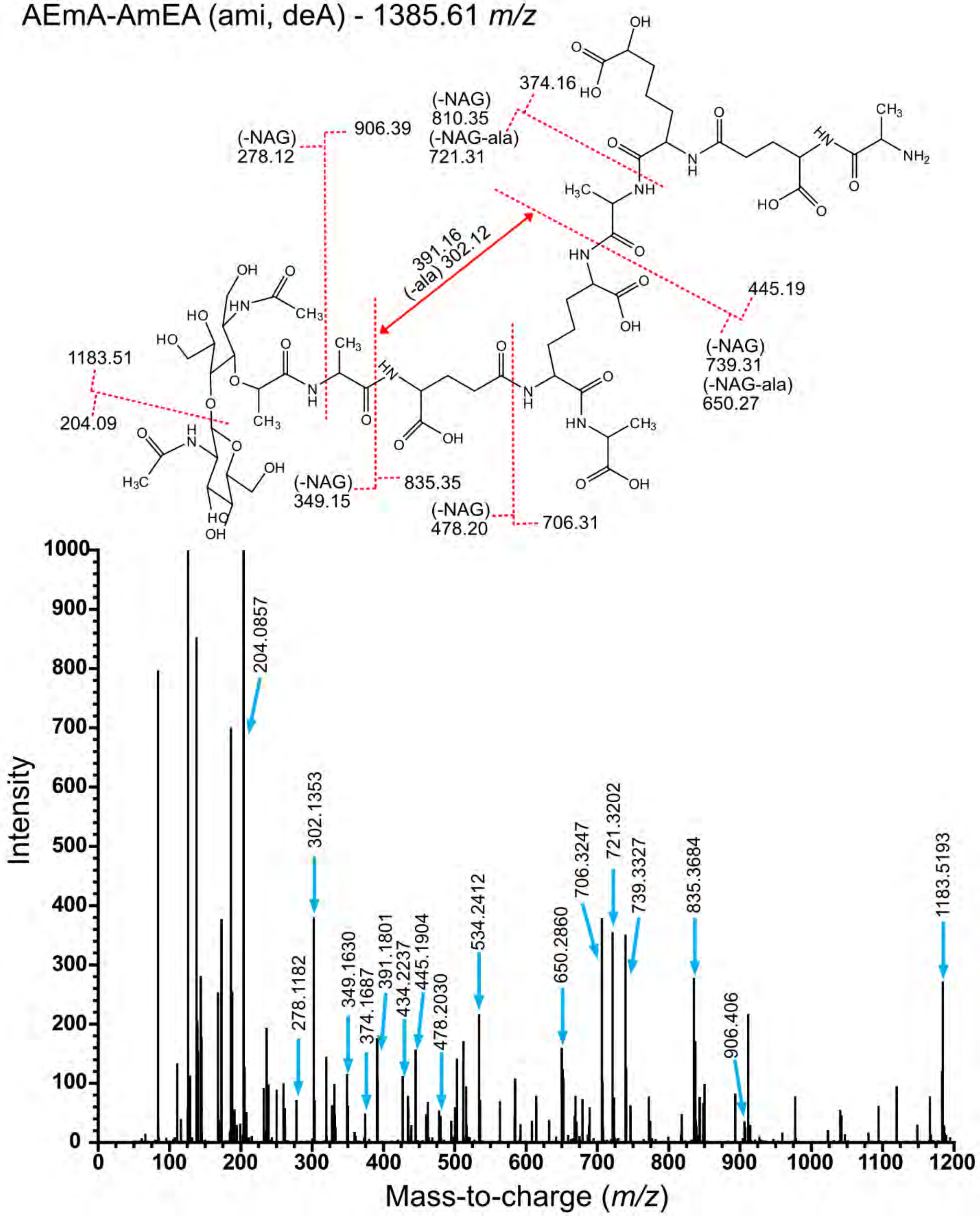

Supplement: FIG S4 [file msystems.00156-22-s0004.pdf]
